# HAP2-Mediated Gamete Fusion: Lessons From the World of Unicellular Eukaryotes

**DOI:** 10.3389/fcell.2021.807313

**Published:** 2022-01-07

**Authors:** Jennifer F. Pinello, Theodore G. Clark

**Affiliations:** ^1^ Department of Cell Biology and Molecular Genetics, University of Maryland, College Park, MD, United States; ^2^ Department of Microbiology and Immunology, Cornell University, Ithaca, NY, United States

**Keywords:** *Tetrahymena thermophila*, *Chlamydomonas reinhardtii*, HAP2/GCS1, membrane fusion, fertilization

## Abstract

Most, if not all the cellular requirements for fertilization and sexual reproduction arose early in evolution and are retained in extant lineages of single-celled organisms including a number of important model organism species. In recent years, work in two such species, the green alga, *Chlamydomonas reinhardtii*, and the free-living ciliate, *Tetrahymena thermophila*, have lent important new insights into the role of HAP2/GCS1 as a catalyst for gamete fusion in organisms ranging from protists to flowering plants and insects. Here we summarize the current state of knowledge around how mating types from these algal and ciliate systems recognize, adhere and fuse to one another, current gaps in our understanding of HAP2-mediated gamete fusion, and opportunities for applying what we know in practical terms, especially for the control of protozoan parasites.

## 1 Introduction

Sexual reproduction was almost certainly present in the last eukaryotic common ancestor (LECA) and continues to be an important if not essential part of the life cycle of organisms ranging from metazoans to single-celled protists (Goodenough and Heitman, 2014; Speijer et al., 2015; Brandeis, 2021). While sex is often cryptic in microbial eukaryotes (Dunthorn and Katz, 2010; Hofstatter and Lahr, 2019), it is readily observed and easy to manipulate in several well-studied model organism species including *Chlamydomonas reinhardtii* and *Tetrahymena thermophila*. Indeed, these simple to grow, genetically tractable systems have yielded important insights into the basic principles underlying gamete-gamete interactions culminating with membrane fusion. This is perhaps best exemplified in recent work on HAP2/GCS1, an ancient gamete fusogen that is now recognized as a catalyst for zygote formation in representative species across all of the major eukaryotic kingdoms of life (Mori et al., 2006; Steele and Dana, 2009; Mori et al., 2015; Speijer et al., 2015).

Elucidation of the role of *HAP2/GCS1* in fertilization began with independent studies in *Arabidopsis thaliana* (Johnson et al., 2004; von Besser et al., 2006), *Lilium longiflorum* (Mori et al., 2006) and *Chlamydomonas reinhardtii* ( Liu et al., 2008 ) demonstrating the necessity of corresponding gene products for male fertility and suggesting their potential role in gamete fusion. Subsequent studies demonstrated that HAP2 is, in fact, a class II (CII) membrane fusogen whose structural features closely mimic those of envelope proteins from Dengue, Zika and related viruses, as well as cell-cell fusion proteins (AFF-1 and EFF-1) from the nematode worm, *C. elegans* which adopt a similar fold (Fédry et al., 2017; Pinello et al., 2017; Valansi et al., 2017). The presence of CII fusogens in eukaryotic cells and the viruses that infect them has interesting and important implications for the origins of sex, the evolution of class II membrane fusogens more generally, and the molecular mechanisms by which HAP2 catalyzes the formation of membrane pores between male and female gametes (Wong and Johnson, 2010; Doms, 2017; Fédry et al., 2017; Pinello et al., 2017; Valansi et al., 2017; Clark, 2018).

The following review addresses our current understanding of gamete recognition, adherence, and fusion in *Chlamydomonas reinhardtii* and *Tetrahymena thermophila*, with an emphasis on the role of HAP2 in membrane fusion and how the HAP2/GCS1 machinery could potentially be exploited to block the transmission of parasitic protists to prevent disease.

## 2 *Chlamydomonas Reinhardtii* and *Tetrahymena Thermophila* as Model Organisms

Owing principally to their ease of growth and facile genetics, *Chlamydomonas reinhardtii* and *Tetrahymena thermophila* have served as key models for studies of eukaryotic cellular and molecular biology since the 1960s (Harris, 2001; Collins, 2012; Orias, 2012; Ruehle et al., 2016; Salomé and Merchant, 2019). A free-living freshwater ciliate that feeds largely on bacteria, *T. thermophila* inhabits lakes and ponds in eastern and central North America. *C. reinhardtii*, on the other hand, is a biciliated unicellular green alga, known principally as a temperate soil dweller but is also found in freshwater ecosystems across a wide geographic range (Sasso et al., 2018).

In the laboratory, *Tetrahymena* and *Chlamydomonas* grow rapidly on inexpensive media in small and large volume cultures, and clonal isolates can be preserved for long-term use (Cassidy-Hanley, 2012; Harris, 2013). More importantly, their sexual cycles can be readily induced and synchronized to generate gametes or mating types that can pair and undergo fertilization in a highly predictable manner. Indeed, gamete fusion in both systems occurs at specific, identifiable regions of mating cells allowing in-depth studies of membrane dynamics during gamete merger. Furthermore, the application of forward and reverse genetics in these systems has made possible the identification of proteins involved in gamete recognition and signaling, membrane adhesion and fusion, and revealed many of the molecular details of fertilization that apply not just to protists but metazoans as well.

Aside from work on HAP2/GCS1 and fertilization more generally, *Tetrahymena* has served as a key model for the study of genome editing (Cheng et al., 2019); stimulus-dependent secretion (Turkewitz, 2004); ciliary and microtubule-based motility (Gibbons and Rowe, 1965; Vale and Yano Toyoshima, 1988; Suryavanshi et al., 2010; Reynolds et al., 2018); ribosome structure and function (Rabl et al., 2011; Wilson and Doudna Cate, 2012); transgenerational inheritance and the role of small RNAs in chromatin dynamics (Couzin, 2002; Liu et al., 2007; Noto and Mochizuki, 2017; Neeb and Nowacki, 2018; Bastiaanssen and Joo, 2021). *Tetrahymena* has also been responsible for major discoveries in the areas of telomere structure and biosynthesis (Blackburn et al., 2006; Jiang et al., 2015); catalytic (self-splicing) RNAs (Herschlag and Cech, 1990; Hedberg and Johansen, 2013); and the role of histone modifications in gene expression (Brownell et al., 1996; Allis and Jenuwein, 2016; Wahab et al., 2020).

Similarly, *Chlamydomonas* has a rich history of important scientific contributions in the areas of photosynthesis and chloroplast structure (Levine and Goodenough, 1970; Engel et al., 2015); ciliary motility, biogenesis and intraflagellar transport (Rosenbaum and Witman, 2002); channelrhodopsins and their applications in optogenetics (Nagel et al., 2003; Zhang et al., 2006; Hegemann and Nagel, 2013); algal biofuel production (Beer et al., 2009; Gimpel et al., 2013); as well as gamete fusion (Ferris et al., 1996; Ferris and Goodenough, 1997; Kurvari et al., 1998; Wang et al., 2006; Liu et al., 2008; Fédry et al., 2017; Zhang et al., 2021).

Genetic strains and other materials including plasmids, BACs, fosmids, educational kits, protocols and other resources are available for *Tetrahymena thermophila* and *Chlamydomonas reinhardtii* through established stock centers at Cornell University, Washington University in St. Louis, and the University of Minnesota (https://tetrahymena.vet.cornell.edu/; https://www.chlamycollection.org/). Additional resources for experimental work in these systems include well maintained genomic and transcriptomic databases (Stover et al., 2012; Xiong et al., 2013; Gallaher et al., 2015, 2018; Blaby and Blaby-Haas, 2017; Sheng et al., 2020). The availability of mRNA expression data for genes that are differentially regulated in resting, lysin-treated, and activated *plus* and *minus* gametes of *C. reinhardtii* (Ning et al., 2013), as well as vegetatively growing, starved and conjugating *T. thermophila* (Miao et al., 2009; Xiong et al., 2013) are particularly relevant to fertilization research.

## 3 Sexual Reproduction in *Chlamydomonas* and *Tetrahymena*: Overview

**FIGURE 1:**
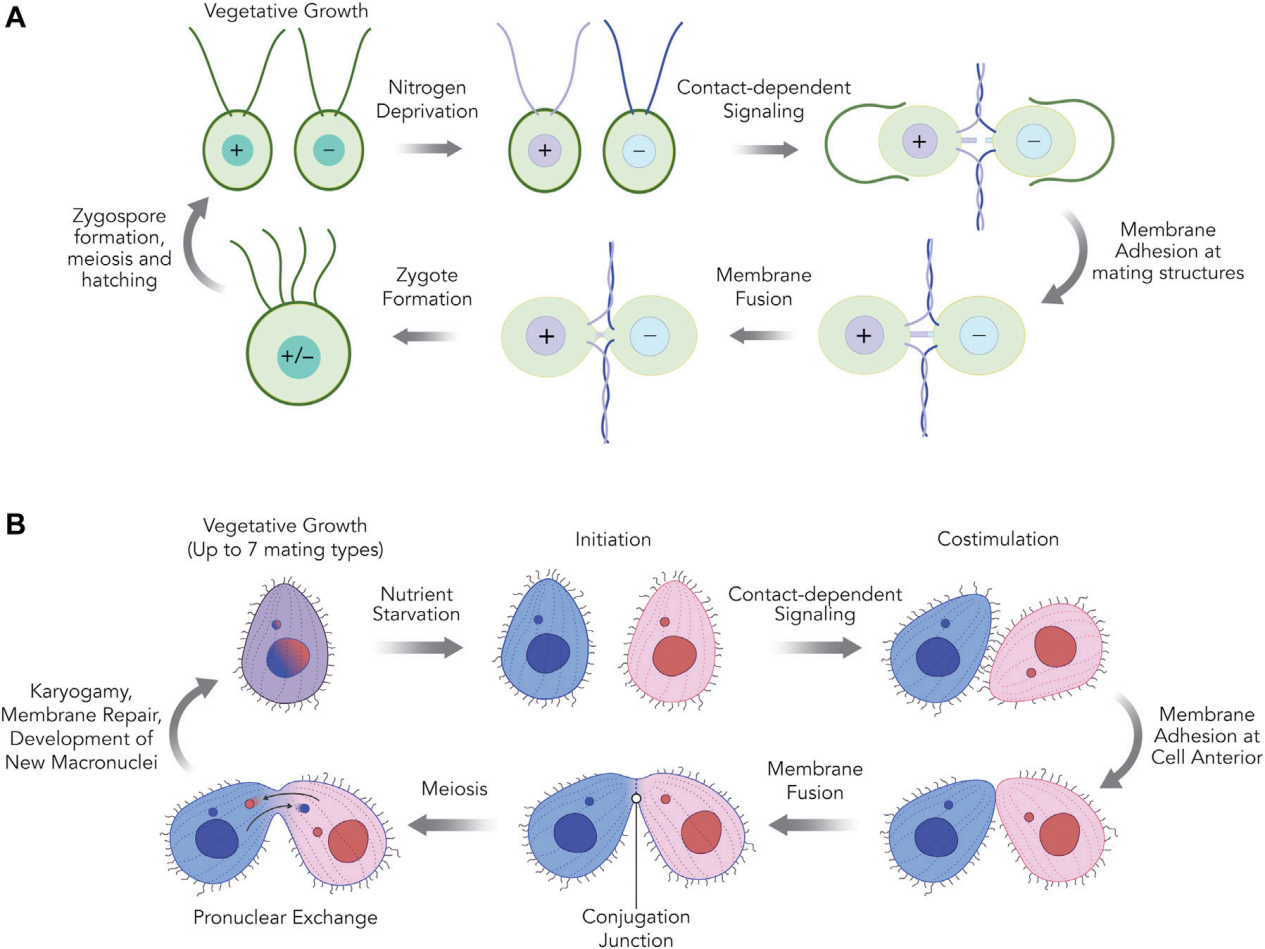
The mating reactions of *Chlamydomonas reinhardtii* **(A)** and *Tetrahymena thermophila* **(B)** cells. **(A)** In *C. reinhardtii*, when asexually dividing haploid cells of *plus* or *minus* mating types are deprived of nitrogen, they undergo gametogenesis. After *plus* and *minus* gametes are mixed, adhesion between *plus* and *minus* cilia stimulate gamete activation; a cyclic AMP-dependent signaling pathway leading to cell wall loss, upregulation of gamete-specific genes, and the maturation of a single membranous protrusion between the two cilia called the mating structure. The tips of the *plus* and *minus* mating structure membranes adhere and fuse quickly, producing a single quadriciliated zygote (∼10 min). Following ciliary resorption, zygotes develop into a resilient dormancy stage with a hard outer cell wall called the zygospore, which is maintained until environmentally favorable (nitrogen-replete) conditions return. Meiosis and hatching of the zygospore produces four haploid progeny cells (two *minus* and two *plus*) capable of vegetative growth. **(B)** In *T. thermophila,* when asexually dividing cells of any of the seven different mating types are deprived of nutrients they undergo “initiation” allowing subsequent sexual interactions. An initiated mating type can undergo conjugation with any of the other six mating types, but not their own. After initiated cells of two different mating types are mixed, physical interactions between the two cells further activate and mature the cells in a contact-dependent stage known as “costimulation,” leading to the upregulation of specific genes and the maturation of a patch of membrane at the anterior end of the cell where mating cells eventually adhere. Membrane adhesion is weak at first but then becomes tight, and by ∼2.5 h cell pairs are firmly joined at a crescent-shaped zone of membrane studded with hundreds of fusion pores called the conjugation junction. Between 5–6 h after mixing, haploid pronuclear products of meiosis made in each of the two partner cells are exchanged across the conjugation junction and fuse to a stationary haploid pronucleus in the partner to create a zygotic nuclear product. These zygotic nuclei undergo further differentiation within their respective cells and at about 10–11 h after mixing, the membrane fusion pores at the conjugation junction are repaired and the two cells separate from one another. Upon return to nutrient-replete conditions, the exconjugant cells immediately divide, generating four progeny “karyonide” cells capable of vegetative growth.

The principal stages leading up to and including gamete fusion in *Chlamydomonas* and *Tetrahymena* are shown in Figure 1. In the case of *C. reinhardtii*, vegetatively growing haploid cells are genetically destined to express either of two mating types, *plus* (+) or *minus* (−) when deprived of nitrogen in the presence of blue light. These conditions bring mating type-specific genes into expression, enabling *plus* and *minus* mating types to eventually pair and then fuse to form zygotes (Ferris and Goodenough, 1997; Goodenough et al., 2007). Pairing begins with interactions between cilia that then triggers cell wall release and the formation of distinct mating structures which protrude from each cell, eventually contacting each other at their distal tips (Cole et al., 2015, 2018). HAP2/GCS1 localizes to the mating structures of *minus* (−) gametes and is essential for initiating formation of a fusion pore that expands into a single contiguous membrane around both cells forming the zygote (Liu et al., 2008, 2015). Subsequent stages of the life cycle, include nuclear fusion (karyogamy) (Ning et al., 2013), zygospore development, meiosis and the formation of four haploid progeny that will divide mitotically when nitrogen is restored (Goodenough et al., 2007).

In the case of *T. thermophila*, sexual reproduction is induced by nutrient starvation, which initiates a program of new gene expression that activates the early stages of mating competence (Bruns and Brussard, 1974; Wellnitz and Bruns, 1979; Xiong et al., 2013). Transient contacts between starved cells of different mating type (referred to as “co-stimulation”) then lead to the upregulation of a further set of genes including those required for adhesion and gamete fusion. Notably, this stage is also accompanied by the remodeling of a region at the anterior of cells where different mating types eventually form tight pairs known as the nuclear exchange or conjugation junction (Pagliaro and Wolfe, 1987; Cole, 2006; Cervantes et al., 2013; Xiong et al., 2013). Following these interactions, HAP2/GCS1 localizes to the junction and catalyzes the formation of a hundred or more individual membrane pores that expand over time to form a lacy curtain separating cells (Cole et al., 2014, 2015). Subsequent stages of sexual development include meiosis, the exchange of migratory haploid pronuclei across the conjugation junction, karyogamy, the development of new macronuclei, restoration of membrane integrity in mating pairs, and separation of progeny cells that will divide mitotically when nutrients are restored. Rather than being sexually dichotomous, *Tetrahymena thermophila* can express up to seven different mating types that are established randomly through genome rearrangements at the mating type (*mat*) locus following sexual conjugation. Individual mating types can be isolated as clonal lines that express a single mating type when sexual reproduction is activated (see below). A given mating type can mate with any of the other six mating types but not with itself.

### 3.1 Acquisition of Mating Competence: Nutrient Deprivation and Cell-Cell Signaling

Acquisition of mating competence in *C. reinhardtii* is induced by suspending vegetatively growing *plus* or *minus* cells in nitrogen-free medium for at least 6 h in the presence of light (Sager and Granick, 1954; Harris, 2009). Nitrogen starvation promotes the formation pre-gametes (Treier et al., 1989; Beck and Acker, 1992) and activates a phototropin responsible for blue light detection (Huang and Beck, 2003). Exposure to light then mediates a signaling cascade responsible for new gene expression and pre-gamete maturation (Pan et al., 1996, 1997).

At the cellular level, nitrogen depletion and gamete maturation are accompanied by a reduction in photosynthetic activity, along with degradation of chloroplasts and cellular ribosomes (Sager and Granick, 1954; Harris, 2009). Transcriptional profiling studies have identified early-, middle-, and late-expressed genes throughout the process that provide molecular markers for the various stages of gametic differentiation (Treier and Beck, 1991; Abe et al., 2004). Interestingly, like *Tetrahymena*, the differentiation of *C. reinhardtii* into mating competent gametes is a reversible process. In the case of *C. reinhardtii,* the re-introduction of nitrogen-replete media in the presence of light will cause cells to revert to a vegetative, asexually dividing state (Sager and Granick, 1954).

Until recently, pheromone-like substances capable of modulating the behavior *C. reinhardtii* gametes had not been identified. In 2019, however, a 23 amino acid amidated peptide that can attract *minus* gametes and repel *plus* gametes was described (Luxmi et al., 2019)*.* This peptide (a short peptide fragment from the cellular protein Cre03.g204500), along with the enzymatic machinery for conversion of peptidylglycine substrates into α-amidated products, was shown to be released from cells via ciliary ectosomes during gametogenesis (Luxmi et al., 2019). While it is not yet clear if this factor is a true pheromone, it is tempting to speculate that bioactive peptides could play such a role in natural aquatic settings where dispersed gametes must find each other to mate.

**FIGURE 2:**
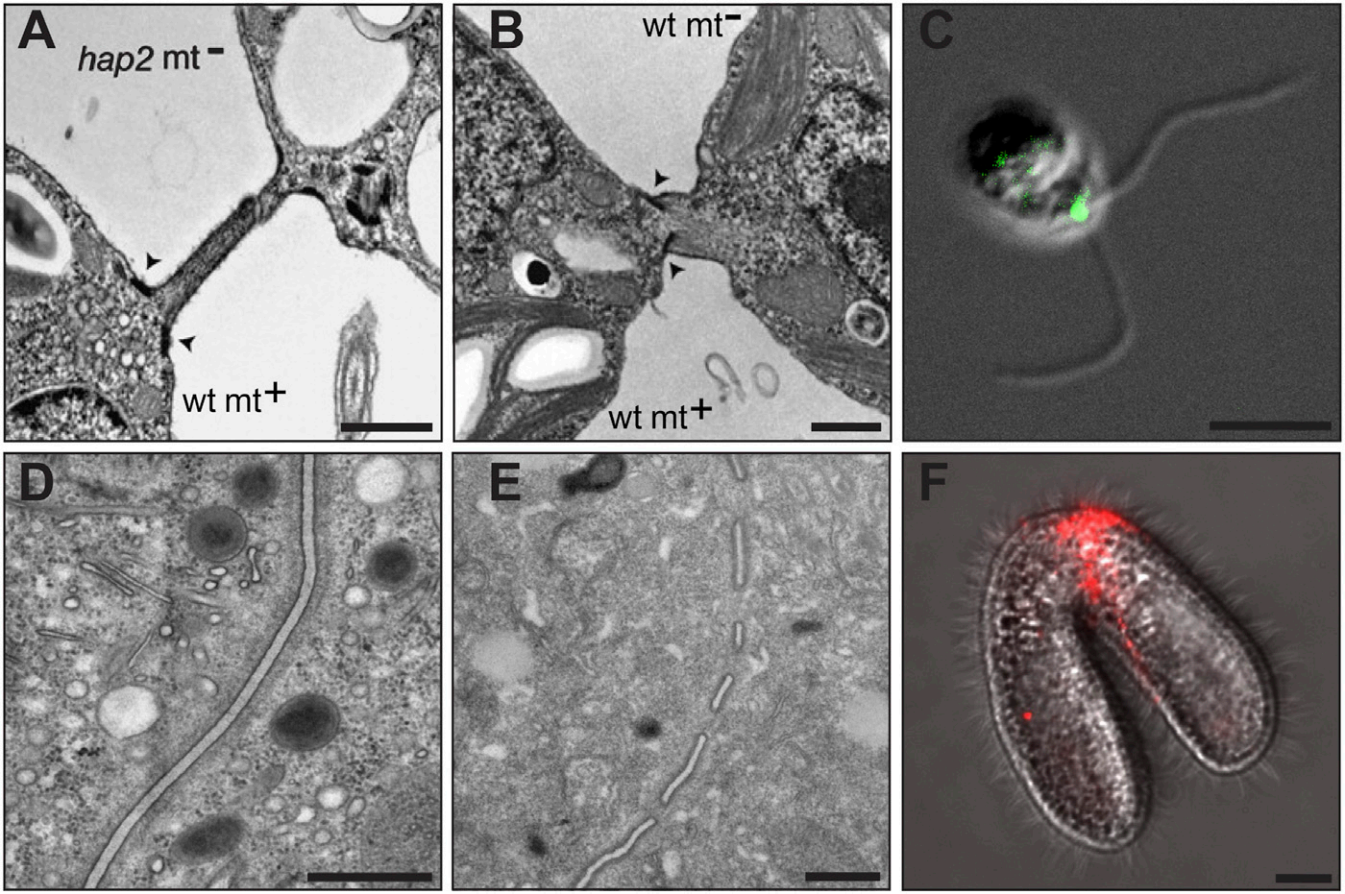
HAP2-mediated membrane fusion in *Chlamydomonas* **(A–C)** and *Tetrahymena* (D–F)*. **(A)** A transmission electron micrograph (TEM) of *Chlamydomonas* gametes showing the adhering mating structures between a *minus* gamete in which the *HAP2* gene was disrupted (*hap2* mt^−^) and a wild type *plus* gamete (wt mt+). Arrowheads point to the electron dense doublet regions at the base of the *plus* gamete mating structure. *Minus* gametes lacking HAP2 adhere to *plus* gametes by the tips of their mating structures but fail to fuse. **(B)** A TEM of wild type *Chlamydomonas plus* and *minus* mating structures immediately after membrane fusion occurs. The fusion pore continues to expand giving way to a single continuous membrane surrounding zygote. **(C)** An immunofluorescence image of a *Chlamydomonas minus* gamete showing a green punctum where HA-tagged HAP2 protein localizes between the two cilia, the site of the *minus* mating structure. **(D)** A TEM showing membranes at the conjugation junction of a *Tetrahymena* mating pair in which both cells have the *HAP2* gene deleted and are unable to form fusion pores. Note the continuous membranes in the center of the image extending from top to bottom. **(E)** A TEM showing multiple membrane fusion pores at the conjugation junction of a wild type mating pair of *Tetrahymena* cells. In wild type pairs, numerous pores, or interruptions are present along the length of the junctional membranes that continue to expand, but never extend beyond the junction itself and are eventually repaired at the conclusion of mating to allow cells to separate. **(F)** An immunofluorescence image of a *Tetrahymena* mating pair showing a red band of signal where HA-tagged HAP2 protein localizes to the conjugation junction between the two mating cells, the site of membrane fusion pore formation. Scale bars are 200 nM **(A–B)**, 5 μMb **(C)**, 500 nM **(D-E)**, and 10 μM **(D)**. *TEMs of interacting *Chlamydomonas* gametes, originally published by Liu et al. in Genes & Development 2008 Apr 15; 22(8):1,051-68 (DOI: 10.1101/gad.1656508) were adapted for this figure and are used here with permission from the authors under license # CC-BY-NC 4.0. TEMs and immunofluorescence image of mating *Tetrahymena* cells, originally published by Cole et al. in Current Biology 2014 Sep 22; 24(18):2,168–2,173 (DOI: 10.1016/j.cub.2014.07.064) were adapted for this figure and are used here with the permission of the journal.

Regardless of a role for putative pheromones in mating behavior, when mature *plus* and *minus* gametes of *C. reinhardtii* are mixed, they quickly adhere through interactions between their cilia (Figure 2). Cultures with large numbers of equally mixed *plus* and *minus* gametes agglutinate and form aggregates. Aggregates eventually sort themselves into individual *plus* and *minus* pairs that then fuse. Ciliary adhesion is mediated by multi-pass transmembrane proteins (agglutinins) on *plus* and *minus* gametes named SAG1 and SAD1, respectively (Goodenough et al., 1978; Hwang et al., 1981; Ferris et al., 2005). Interactions between these proteins trigger protein kinase- and kinesin-2 dependent activation of a ciliary adenylyl cyclase, followed almost immediately by a near 20-fold increase in intracellular levels of cAMP (Zhang et al., 1991; Saito et al., 1993; Pan and Snell, 2002; Wang et al., 2006; Snell and Goodenough, 2009).

Elevation of intracellular cAMP is required for all subsequent morphological changes associated with gamete activation, including the enlargement of ciliary tips, cell wall loss, and the emergence of mating structures (Pasquale and Goodenough, 1987). Although SAG1 and SAD1 are detectable on cilia of naive gametes, the majority is present on plasma membranes. Interestingly, SAG1 has been shown to be recruited from the cell body to cilia in a microtubule-dependent fashion (Ranjan et al., 2019) and appears to be shed from cilia in association with ectosomes (Cao et al., 2015; Wood and Rosenbaum, 2015). Addition of purified SAG1-containing ectosomes to cultures of naive *minus* gametes causes isoagglutination and gamete activation in the absence of a mating partner. Similarly, addition of exogenous dibutyryl cAMP, together with inhibitors of cyclic nucleotide phosphodiesterase, bypasses the requirements for ciliary recognition and can alone stimulate the morphological changes that accompany gamete activation (Pijst et al., 1984; Pasquale and Goodenough, 1987).

As noted above, removal of the cell wall, a prerequisite for gamete fusion, is also triggered by elevated intracellular cAMP. Cell wall lysis is catalyzed by a secreted zinc metalloproteinase termed gametic lysin (Claes, 1971; Schlösser et al., 1976; Buchanan and Snell, 1988) that is stored in an insoluble inactive state in the periplasm (Claes, 1977; Matsuda et al., 1978; Buchanan et al., 1989). Upon agglutination and elevation of intracellular cAMP, the enzyme becomes activated through cleavage by a serine-like protease resulting in cell wall dissolution (Snell et al., 1989; Luxmi et al., 2018). Interestingly, the cell wall of *Chlamydomonas* is composed of hydroxyproline-rich glycoproteins rather than cellulose (Horne et al., 1971; Adair and Mecham, 1990; Woessner and Goodenough, 1992; Ferris et al., 2001). Likewise, many *Chlamydomonas* gamete-specific proteins involved in recognition and adhesion (*e.g.* SAG1, SAD1, and MAR1) contain hydroxyproline-rich repeats (Adair, 1985), although it is unclear whether any of these are substrates for gametic lysin.

In addition to control by cAMP, experiments using the calcium ionophore A23187 have demonstrated a role for Ca^++^-signaling in cell wall dissolution. In these studies, A23187 by itself triggered cell wall lysis of both *plus* and *minus* mating types in isolation (Claes, 1980). Later studies showed that lidocaine (in a manner reversible by addition of Ca^2+^ and Mg^2+^) inhibited cell wall loss without interfering with ciliary agglutination or tip activation (Snell et al., 1982). While the pharmacology of calcium channels in *C. reinhardtii* is still under scrutiny (Liang and Pan, 2013), these findings suggested that elevation of cAMP may alter channel activity in ciliary membranes allowing Ca^++^ entry, secretion of stored gametic lysin and cell wall dissolution.

Following cell wall removal, membrane protuberances known as mating structures form at the anterior of cells between the cilia. Formation of the mating structures along with subsequent membrane events associated directly with gamete fusion in *C. reinhardtii* are described below (*Membrane dynamics at sites of gamete fusion*).

In *Tetrahymena thermophila*, nutritional starvation is achieved by washing cells of different mating types into low ionic strength buffers for 70–75 min at 30°C (Wellnitz and Bruns, 1979). The period of “initiation” prior to allorecognition (that is, physical contacts between cells of different mating types) is accompanied by up-regulation of >200 genes (Xiong et al., 2013) and is inhibited by elevated buffer concentrations (Wellnitz and Bruns, 1979). Once initiation is complete, cells of different mating types can be combined into a single flask, allowing contacts between cells, effectively synchronizing the later stages of sexual development.

Transient interactions between cells (referred to as “co-stimulation”) triggers a new round of RNA and protein synthesis involving up-regulation of >1,800 genes (Xiong et al., 2013), many of which, including *HAP2/GCS1,* turn on almost immediately after cells are combined (Cole et al., 2014). While the signaling pathways activated during this stage have yet to be defined, several factors come into play during co-stimulation. First, a non-mating type specific substance is continuously released from cells in response to nutritional starvation and is necessary for different mating types to advance to full mating competence during the process of co-stimulation (Adair et al., 1978; Wolfe et al., 1979). This material is relatively heat stable and non-dialyzable, although its identity and role in cell-cell signaling are unknown.

Along with this “soluble” factor, different mating types must undergo multiple direct contacts between cells over a period of at least 20 min to activate the downstream events required for tight (that is, mechanically stable) pairing, which begins approximately 1 h after starved cells are mixed (Brown et al., 1993). Interestingly, as cells become activated, they initially form weak, transient pairs that can be either homotypic (between cells of the same mating type) or heterotypic (between cells of different mating type). Only heterotypic interactions provide the signals necessary to fully activate cells. Nevertheless, the fact that weak homotypic pairing occurs in the lead-up to conjugation suggests that cells produce both (mating type) specific and non-specific adhesive molecules as they become activated (Cole, 2016).

While the signaling pathways triggered by collisions between different mating types of *T. thermophila* are still unknown, there is substantial indirect evidence that, as in the case of *C. reinhardtii*, activation is initiated through contacts between cilia (Love and Rotheim, 1984; Wolfe et al., 1993). First and foremost, deciliated cells are unable to pair (Wolfe et al., 1993). Experiments to explore whether purified cilia from one mating type can induce mating competence in cells of a different mating type have nevertheless failed because free cilia are phagocytosed and act as a protein source, eliciting starved cells to exit the sexual cycle and begin vegetative growth (Wolfe et al., 1993).

Irrespective of the role of cilia in contact-dependent signaling, the weak homotypic interactions that occur early after starved cells are combined, eventually give way to more numerous and stable interactions between cells of different mating types. Such interactions occur primarily along the ventral surfaces of cells, anterior to the oral apparatus where mating cells eventually form “tight” pairs along a specialized region known as the conjugation junction (Cole, 2006, Cole, 2016) (Figure 1). The lead up to tight pairing is accompanied by major structural changes at the cell cortex where the junction eventually forms including loss of dense core secretory granules, alveolar membranes, cilia and ciliary basal bodies, along with changes in shape at the cell anterior from pointed to slightly blunt (Wolfe and Grimes, 1979). More global changes in overall cell shape (e.g., shortening along the longitudinal axis) have also been noted (Fujishima et al., 1993).

Coincident with these structural alterations, concanavalin-A (Con-A) binding receptors at the plasma membrane become concentrated at the cell anterior, a phenomenon known as Con-A “tipping” (Wolfe and Grimes, 1979; Wolfe and Feng, 1988). The addition of Con-A to live cells during “co-stimulation” blocks the formation of mating pairs suggesting that glycosylated receptors (presumably, membrane glycoproteins) play a role in adhesion. The redistribution of Con-A receptors to the nascent conjugation junction along with the changes in cell shape that accompany mating would also argue for the involvement of the cytoskeleton in the events leading up to adhesion and membrane pore formation. Coincidentally, Con-A has been shown to block the mating reaction in *Chlamydomonas* as well, although by an entirely different mechanism that involves an inhibition of cell wall dissolution (Claes, 1975), possibly through lectin binding to gametic lysin (Snell et al., 1982).

### 3.2 Mating Type Determination

At the genetic level, mating types of *Chlamydomonas* and *Tetrahymena* are governed by genes at specific chromosomal regions known as the mating type loci. In *C. reinhardtii*, the mating type locus encompasses a genetically complex, 0.2–0.4 Mbp region on chromosome six that is rearranged between the two mating-type haplotypes and orchestrates the expression of genes for gamete recognition, adhesion, and fusion, as well as genes involved in sporulation, mitochondrial and chloroplast inheritance (Goodenough et al., 1995; Ferris et al., 2002; De Hoff et al., 2013). Underlying control of these genes is the RWP-RK transcription factor, Minus Dominance, or *MID*, a master regulator expressed in *minus* mating type cells following cAMP-dependent activation (Ferris and Goodenough, 1997; Lin and Goodenough, 2007).

MID is only present at the mating type locus of minus cells and exerts its control by suppressing *plus* gamete-specific developmental programs and stimulating the expression of *minus* gamete-specific genes such as HAP2 and MAR1 (see below) that lie outside the locus itself (Ferris and Goodenough, 1997; Lin and Goodenough, 2007). For example, the presence of MID blocks the expression of *plus* gamete specific ciliary agglutinin SAG1, but induces the expression of the *minus* gamete-specific agglutinin SAD1 (Sekimoto, 2017). In *minus* cells, mutations of MID promote differentiation into infertile “pseudo-*plus*” gametes, while the forced expression of MID in *plus* cells leads to their differentiation into minus gametes (Ferris and Goodenough, 1997; Goodenough and Heitman, 2014). As with C. *reinhardtii*, ectopic expression of MID in female (*plu*s) gametes of the related alga, V. *carteri*, leads to a pseudo-male gametic phenotype (Geng et al., 2014), and MID orthologs can be functionally substituted between different species of algae (Geng et al., 2018). These findings demonstrate a conserved role of MID in algal mating type determination, and indicate that plus/female gamete differentiation is the default state in these systems (Goodenough and Heitman, 2014; Coelho and Umen, 2021).

Interestingly, MID homologs have been identified in other protists (*Dictyostelium discoideum* and *Entamoeba histolytica*) as well as in *land plants* (Riaño-Pachón et al., 2008; Blanc-Mathieu et al., 2017; Yamazaki et al., 2017; Geng et al., 2018). In contrast with algae, however, RWP-RK transcription factors in plants specify female gamete differentiation, while Myb transcription factors (DUO1 and DUO3) control spermatogenesis and upregulation of male gamete-specific transcripts such as HAP2 (Borg et al., 2011; Higo et al., 2018; Hisanaga et al., 2019).

Finally, aside from regulation at the transcriptional level, mating type ratios in *Chlamydomonas* are maintained through differences in sequence organization at the mating type loci of *plus* and *minus* cells, which have a suppressive effect on recombination within this region of chromosome 6 (Goodenough et al., 2007; De Hoff et al., 2013). As a consequence, the zygote is always heterozygous diploid with respect to the mating type locus and gives rise to four meiotic progeny at fixed ratios (*viz*., 2:2) of *plus* and *minus* cells that can easily be separated after hatching (Goodenough et al., 2007).

In the case of *Tetrahymena*, mating types are also specified by proteins encoded at the mating type locus (*mat*) (Nanney and Caughey, 1953; Orias et al., 2017). Nevertheless, rather than being inherited in a Mendelian fashion, mating types of *T. thermophila* are determined randomly and independently in cell progeny after fertilization has occurred (Cervantes et al., 2013; Orias et al., 2017). The molecular basis of mating type determination in *Tetrahymena* was established in landmark work by investigators at the University of California, Santa Barbara and the Wuhan Academy of Hydrobiology in 2013 (Cervantes et al., 2013). Briefly, the mating type genes encode pairs of transmembrane proteins designated, MTA and MTB, that are expressed upon starvation. While sexually mature vegetatively growing cells have the potential to express only a single mating type specified by one *MTA/MTB* gene pair in somatic macronuclei, germline micronuclei of inbred *T. thermophila* strains contain several incomplete *MTA*/*MTB* gene pairs (five or six depending on the micronuclear *mat* allele), specifying different mating types and organized in a tandem array at the *mat* locus. After fertilization and karyogamy, developing macronuclei undergo a series of immunoglobulin-like genome rearrangements that give rise to one functional *MTA/MTB* gene pair at the *mat* locus, with each of the four progeny cells (karyonides) from a given mating having the potential to generate a different functional gene pair on a random basis (Cervantes et al., 2013).

The *MTA* and *MTB* genes of *T. thermophila* are distantly related and gave rise to paralogs in different *Tetrahymena* species presumably through gene duplication and mutational drift (Orias et al., 2017; Yan et al., 2021). Nevertheless, each of the seven MTA and MTB proteins have variable N-terminal extracellular domains with two tandem cysteine-rich furin-like repeat motifs of unknown function, along with C-termini containing five membrane-spanning helices. Mating type specificity most likely resides in the predicted extracellular portion of MTA and MTB, which comprise at least two thirds of each protein. The precise details of allorecognition remain to be established, and it is not known whether the MTA and MTB proteins act in concert (that is, as heterodimers) or independently, or whether they mediate the same or different aspects of recognition and adhesion, however the deletion of the corresponding genes impairs the ability of cells to pair or form progeny (Cervantes et al., 2013).

While mating type selection is a prerequisite for sex in *T. thermophila*, there is at least one additional step necessary for cells to achieve full sexual maturity. At some point after conjugation, vegetatively growing cells reach a stage termed “adolescence” where they can mate successfully with cells that are fully sexually mature, but not with other “adolescent” cells despite their ability to express compatible mating type proteins (Rogers and Karrer, 1985). Why this is true has yet to be determined, although it may be that these adolescent cells are unable to respond to, or deliver signals to other adolescent cells during the process of “co-stimulation” (Rogers and Karrer, 1985). Apparently, adolescent cells must undergo additional cell divisions to reach full sexual maturity.

### 3.3 Membrane Dynamics at Sites of Gamete Fusion

Despite similarities in the mechanisms that trigger mating competence in *Chlamydomonas* and *Tetrahymena*, the membrane events associated with gamete adherence and fusion in these systems are quite different. In *Chlamydomonas*, membrane fusion is initiated at the tips of so-called, mating structures (also known as “fertilization tubules”), microvillus-shaped protrusions of the plasma membrane at the anterior ends of *plus* and *minus* gametes between the two cilia (Weiss et al., 1977) (Figures 2A,B). In naive *plus* gametes, the mating structures bud from an area of electron density (referred to as “doublets”) just beneath the plasma membrane and extend ∼3 μM from the membrane at their maximal length (Cavalier-Smith, 1975; Goodenough and Weiss, 1975; Triemer and Malcolm Brown, 1975). *Plus* mating structures are supported by actin filaments *in vivo* and can be isolated from mechanically disrupted cells by differential centrifugation through sucrose and Percoll density gradients (Detmers et al., 1985; Wilson et al., 1997). *Minus* mating structures are more diminutive (∼1 μM in length), have no obvious cytoskeletal support, and bud from an electron dense patch of membrane with no underlying doublet zone (Weiss et al., 1977; Goodenough et al., 1982). The extracellular surfaces of activated *plus* and *minus* mating structures appear to be coated with a fringe of proteinaceous material visible by transmission electron microscopy (Goodenough et al., 1982).

Interactions between the cilia bring the mating structures of each cell into close contact. Once contact occurs, membranes at the tips of the mating structures adhere and fuse. The cytoplasmic bridge linking the cells then shortens rapidly and the gametes themselves transition from a face-to-face to a side-to-side orientation as they continue to fuse laterally from anterior to posterior until they are completely merged (Snell and Goodenough, 2009). In mass cultures, the reaction is highly synchronous and 1:1 mixtures of *plus* and *minus* gametes complete fusion within ∼10 min.

Recently, the FUS1-MAR1 receptor pair essential for mating structure adherence was identified. FUS1 is expressed only in *plus* gametes (Misamore et al., 2003), and the mating structures of *fus1 plus* gamete mutants (whose FUS1 gene is disrupted) are incapable of adhering to those of *minus* gametes despite normal interactions between cilia (i.e., agglutination) and activation (Ferris et al., 1996; Misamore et al., 2003). Transmission electron microscopy has shown that *fus1* gametes lack the proteinaceous fringe on the surface of their activated *plus* mating structures (Goodenough et al., 1982). Consistent with this, immunofluorescence localization studies have shown that FUS1 protein redistributes along the entire surface of the *plus* gamete mating structure when cells become activated (Misamore et al., 2003). The *FUS1* gene encodes an 823 amino acid glycoprotein with a long N-terminal extracellular domain, a single transmembrane helix, and a short cytoplasmic tail (Ferris et al., 1996). The extracellular region is characterized by seven immunoglobulin-like domains and bears a strong resemblance to the modeled protein structure of the plant sperm gamete adhesion protein, GEX2 (Mori et al., 2014; Pinello et al., 2021). This structural resemblance suggests that FUS1 and GEX2 may be members a conserved family of gamete adhesion proteins shared across green organisms.

Co-immunoprecipitation studies have shown that MAR1 and FUS1 directly interact through their respective ectodomains. Furthermore, disruption of the *MAR1* gene (*mar1*) in *minus* gametes prevents the adherence of mating structures and subsequent fusion in crosses with wild type *plus* gametes but has no effect on ciliary adhesion and gamete activation.

A transgene encoding a FLAG-tagged version of MAR1 can rescue adherence of mating structures and gamete fusion when introduced into *mar1 minus* cells. Furthermore, expression of the same construct can rescue adherence of mating structures when introduced into *minus* mutants carrying disruptions in both *mar1* and *hap2*, demonstrating a key role for MAR1 in membrane adhesion *per se* (Pinello et al., 2021). Immunofluorescence localization studies indicate that MAR1 is only expressed in *minus* gametes and localizes to sites where the mating structures appear in both naive and activated cells.

The *MAR1* gene encodes a 1018-residue, single-pass transmembrane protein with a long cytoplasmic tail. Orthologs are present in only a few closely related algal species suggesting that MAR1 is a lineage-specific adhesion protein (Pinello et al., 2021). Interestingly, the MAR1 ectodomain contains a proline-rich region with five repeating “PPSPX” motifs that are seen in other *Chlamydomonas* hydroxyproline-rich glycoproteins such as SAG1 and SAD1 (Ferris et al., 2005). Notably, aside from its interactions with FUS1, MAR1 also interacts with the gamete fusogen, HAP2. Antibody pull-down studies have demonstrated that FLAG-tagged MAR1 can associate with HAP2-HA, and MAR1 is required for proper expression and localization of HAP2 on the mating structures of *minus* gametes as shown by immunofluorescence microscopy (Pinello et al., 2021). These interactions suggest that MAR1 and other lineage-specific gamete adhesion proteins may act as gatekeepers for the gamete membrane fusion reactions in *C. reinhardtii* and other species which rely on HAP2 for fertilization.

Aside from FUS1 and MAR1, mutant studies indicate that additional proteins may be involved in gamete adherence and fusion in *C. reinhardtii*. For example, a temperature sensitive *minus* gamete mutant, *gam10*, has been shown to allow adhesion between mutant (*minus*) and wild type (*plus*) cells via their mating structures but is blocked in gamete fusion (Forest, 1983). The *gam10* mutation is not *HAP2* since the *HAP2* gene is intact and also expressed in *gam10* cells (Liu et al., 2010). However, a mutant cell line with a *minus* phenotype similar to that of *gam10* has recently been identified that is defective in a gene with putative involvement in the 5-deoxystrigol biosynthetic pathway (Aksoy et al., 2021). Further characterization of this strain will be necessary to define a possible relationship with *gam10*, along with a potential role for the 5-deoxystrigol biosynthetic pathway in gamete adherence and/or fusion. In other studies, a disruption in *MID*, the master regulator of mating type determination, has suggested a role for additional proteins in gamete adhesion and/or fusion. In this case, the *imp11 minus* cell line, which is defective in *MID*, defaults to an infertile pseudo-*plus* phenotype. These cells lack the *plus* mating type locus (along with *FUS1*) and produce mating structures that are unable to adhere or fuse to wild type *minus* gametes. Interestingly, ectopic expression of a *FUS1* transgene in the *imp11* strain rescued the ability of these cells to undergo mating structure adhesion with wild type *minus* cells (Goodenough et al., 1982; Galloway and Goodenough, 1985; Ferris et al., 1996) but gamete fusion was still impaired (rapid fusion only occurred in response to pH shock) (Ferris and Goodenough, 1997; Misamore et al., 2003). Since the wild type *minus* gametes in those crosses expressed functional versions of HAP2 and MAR1, some uncharacterized protein(s) specified by the *plus* mating type locus (which is not present in the *imp11* mutant) likely contributes to efficient gamete fusion in *Chlamydomonas*.

Finally, while the mating structures of *plus* gametes contain abundant actin filaments, *plus* cells pretreated with cytochalasin D can be activated and produce mating structures that lack F-actin when mixed with *minus* gametes. These actin-less *plus* mating structures make contact with the tips of the mating structures of *minus* cells but membrane fusion is strongly inhibited in these pairs (Mesland et al., 1980; Goodenough et al., 1982; Detmers et al., 1983). Additionally, the *ida5* strain of *C. reinhardtii*, which contains a nonsense mutation in the sole actin gene of *C. reinhardtii* resulting in a large deletion towards the actin C-terminus, also shows greatly reduced gamete fusion (Kato-Minoura et al., 1997). These findings strongly suggest that filamentous actin within the *plus* gamete mating structure facilitates *Chlamydomonas* cell-cell adhesion and/or fusion and are certainly consistent with now growing evidence for the involvement of F-actin in cell-cell fusion in metazoan cells (Kim and Chen, 2019; Chan et al., 2020).

In the case of *Tetrahymena*, different mating types adhere and fuse at a specialized region near the anterior of cells known as the conjugation junction (Cole, 2006). The junction itself lacks structures normally associated with the cell cortex and instead becomes an organizing center for membrane remodeling events including the formation of hundreds of HAP2-dependent fusion pores (Cole et al., 2015, 2018). When viewed *en face*, the boundaries of the conjugation junction take the shape of an inverted heart, or chevron roughly 8–10 μm in diameter. The apposed membranes on either side are separated at a uniform distance of ∼40 nm (Figure 2) and appear to be supported by proteinaceous scaffolds on their cytosolic face (Wolfe, 1982; Cole et al., 2015).

Initially, junctional membranes are planar and continuous, but as pairs begin to form, out-pocketings appear on both membranes extending into the extracellular space and towards the apposed membrane on either side of the junction. These protuberances (which are roughly the diameter of the mating structures of *Chlamydomonas*, viz., ∼50 nm), eventually fuse with the apposed membrane creating pores along the length of the junction that connect the two cells (Cole et al., 2015). Over time, the pores expand laterally, eventually forming a network, or curtain of membrane tubules as their advancing fronts approach each other (Wolfe, 1982; Cole, 2006). The fact that membrane protuberances which mark the sites of membrane fusion are generated from both cells of a mating pair would clearly argue that the initiation of pore formation is not restricted to a single mating type in this species. That notion is strongly supported by the observation that *HAP2/GCS1* is expressed in all seven mating types of *T. thermophila* as well (see below).

Conventional electron microscopy, freeze fracture and 3-D reconstructions of cryopreserved sections (electron tomography) have offered spectacular views of junctional membranes during pore formation and expansion, along with insights into the complex nature of these processes (Wolfe, 1982; Orias et al., 1983; Cole et al., 2015, 2018). For example, following the initiation of pore formation, small vesicles or tubules are released into the extracellular (luminal) space at sites immediately adjacent to nascent pores, only to be enveloped by membrane clefts or folds extending into the lumen from sites more distal to the pores (Cole et al., 2014, 2015). These membrane high jinks result in the formation of what appear to be multivesicular bodies that are reclaimed into the cytoplasm with the overall process contributing to, if not underlying, pore expansion (Cole et al., 2015). Adding to the complexity, an entirely separate trans-junctional membrane reticulum (presumably an extension of the smooth endoplasmic reticulum), invades the pores from either side, coming in close proximity to their borders (Cole et al., 2015). The close associations between the trans-junctional membrane reticulum and dynamic pore structures has suggested that lipid exchange between the two membrane systems may occur in order to support pore formation and expansion (Cole et al., 2015).

In this regard, it is worth noting that mass spectrometric imaging studies of mating cells indicate that membranes at the conjugation junction are depleted in the abundant cylindrical lipid, phosphatidyl choline, and are enriched in cone-shaped lipids such as 2-aminoethylphosphonolipid compared with membranes on the cell body (Ostrowski et al., 2004). This finding is clearly consistent with the large numbers of pores at the conjugation junction and their intrinsic membrane curvature. Somewhat paradoxically however, kinetic studies also suggest that depletion of phosphatidyl choline at the junctional membranes occurs after most cells have formed pores, which would argue that alterations in membrane lipids are not a driving force in the initiation of pore formation (Kurczy et al., 2010).

Although HAP2/GCS1 is almost certainly the catalyst for cell-cell fusion during fertilization in *T. thermophila* (see below), the complexities of pore formation and expansion revealed in ultrastructural studies clearly suggest that other proteins are involved in the initiation and resolution of membrane pores in this system. To identify such proteins, a variety of innovative approaches have been employed. Ethanol fixation and mechanical disruption of mating pairs by sonication has yielded structures remarkably similar in size and shape to the conjugation junction (Cole et al., 2008). Using a proteomics-based approach, it was possible to identify as many as 15 proteins associated with these structures (Cole et al., 2008), some of which were likely contaminants, and some, bona fide constituents of the conjugation junction including “fenestrin”, a 64 kDa protein that had been linked to the nuclear exchange junction in previous work (Nelsen et al., 1994), and cytoskeletal proteins (epiC; α- and β-tubulin) that had also been localized to the conjugation junction using immuno-labeling techniques (Orias et al., 1983; Williams et al., 1987; Gaertig and Fleury, 1992; Williams et al., 1995).

Another fruitful approach towards identifying proteins involved in fertilization in the *Tetrahymena* system is illustrated by studies on Zfr1, a zinc-finger protein that appears to have a role in cell-cell pairing (Xu et al., 2012). *ZFR1* was identified as a member of a network of genes that is upregulated when starved cells of different mating types are mixed. Tagging and over-expression of the corresponding protein showed that the *ZFR1* gene product localizes to the conjugation junction. Consistent with its lack of expression during vegetative growth, deletion of the *ZFR1* gene had no apparent effect on mitotically growing cells (Xu et al., 2012). By contrast, crosses between nutritionally starved *ΔZFR1* knockout strains were found to be capable of forming pairs, but failed to complete normal sexual development (Xu et al., 2012). On closer inspection, *ΔZFR1* knockout pairs came apart earlier during the sexual cycle and were more sensitive to mechanical disruption when compared to wild type pairs. This unstable pairing phenotype clearly suggests that the corresponding gene product plays some role in the adherence of mating cells, and while Zfr1 is not predicted to be a membrane protein, it does contain a hydrophobic C-terminus and may traffic through the Golgi apparatus based on localization studies (Xu et al., 2012). Of course, an indirect role for Zfr1 in pair stability through an effect on other proteins cannot be ruled out. Regardless of the precise role of Zfr1 in membrane adhesion, reverse genetic approaches involving deletion of genes that are upregulated during conjugation have proven to be extremely informative in the case of both *ZFR1* and *HAP2* and could easily be applied to identify other proteins that play a role in membrane adhesion and/or pore formation in *Tetrahymena*.

Finally, less systematic approaches have allowed the identification of a number of other proteins that localize to the conjugation junction at different time points in mating and may play important roles in fertilization in the *Tetrahymena* system. Such proteins include, Cda13p, a small membrane protein believed to have a role in membrane trafficking that also localizes to a ring associated with the junction in the period immediately before and just after pronuclear exchange (Zweifel et al., 2009); BLT1, a β-tubulin multigene family member which localizes to micronuclei and micronuclear meiotic spindles of conjugating cells that transiently decorates the nuclear exchange junction (Pucciarelli et al., 2012); and TCB25 (Tcb2), a calcium-binding protein thought to play a role in pronuclear exchange (Hanyu et al., 1995; Cole et al., 2018).

### 3.4 HAP2-dependent Gamete Fusion

Genetic screens for male sterility in *Arabidopsis thaliana* identified *hap2* as one of 32 haploid-disrupting (*hapless*) genotypes that define pollen grain development and/or pollen tube growth and guidance in this species (Johnson et al., 2004). In parallel studies, transcriptional profiling of mRNAs expressed at different stages of pollen development in *Lilium longiflorum*, identified a gene, designated *GCS1*, that was specifically upregulated in generative cells, the precursors of sperm (Mori et al., 2006). *HAP2* and *GCS1* were homologs, and targeted gene disruptions of *HAP2* in *Arabidopsis* established a role for the corresponding gene product in fertilization and suggested a possible function in gamete recognition/activation, sperm-egg attachment, or sperm-egg fusion (Johnson et al., 2004; von Besser et al., 2006; Mori et al., 2006). Independently, studies in *Chlamydomonas reinhardtii* and the malarial parasite *Plasmodium falciparum,* solidified the importance of HAP2/GCS1 in fertilization and extended the work in plants by demonstrating a functional role for HAP2 at a step after gamete adhesion (most likely fusion) in two widely diverged protists (Liu et al., 2008). Furthermore, the presence of *HAP2/GCS1* homologs in species outside of plants argued persuasively that its function was conserved across a broad range of taxa (Mori et al., 2006; Liu et al., 2008; Steele and Dana, 2009; Wong and Johnson, 2010).

In *Chlamydomonas*, *minus* gametes with a disruption in the *HAP2* gene (*hap2*) underwent normal ciliary adhesion, gamete activation, and mating structure adhesion when mixed with *plus* gametes but failed to complete fertilization and form quadriciliated zygotes (Liu et al., 2008). Importantly, HAP2/GCS1 localized precisely to the region of *minus* cells where gamete fusion occurs (namely the *minus* mating structure) and ultrastructural studies demonstrated an inability of *plus* and *minus* mating structures to fuse in crosses between wild type *plus* and *hap2*-disrupted *minus* strains (Liu et al., 2008) (Figures 2A–C). Similar assays in *Plasmodium berghei* found that HAP2/GCS1 was expressed and localized over the entire plasma membrane of male gametes (consistent with indiscriminate sites of gamete attachment and membrane fusion in this species), while mutant cells deficient in HAP2 were able to attach but were unable to fuse with female gametes (see below). Subsequent studies in *C. reinhardtii* demonstrated that the HAP2 protein, along with the fertilization-essential membrane proteins, FUS1 and MAR1, were rapidly degraded after cell-cell fusion, showing tight regulation of the gamete membrane fusion machinery in a possible block to polygamy (Johnson, 2010; Liu et al., 2010; Pinello et al., 2021).

Following this work, studies in *Tetrahymena* reinforced the idea that HAP2 functions downstream of gamete membrane attachment, but with an interesting twist (Cole et al., 2014). *T. thermophila* has seven sexes/mating types raising an interesting question as to how a “male” gamete-specific fusion factor functions in an organism that is, for all intents and purposes, sexually ambiguous. Studies by Cole et al. demonstrated that *HAP2* was expressed in all seven mating types of *T. thermophila*, and that fertilization and membrane fusion were completely blocked only when *HAP2* was disrupted in both cells of a mating pair (Cole et al., 2014). As shown in Figure 2, different mating types lacking *HAP2* adhered to one another along their entire junctional interface but failed to form fusion pores (Figure 2D). In wild type crosses, however, characteristic fusion pores formed at regular intervals along the junctional membranes creating cytoplasmic bridges between mating cells (Figure 2E). Localization studies at the light (Figure 2F) and electron microscopic levels demonstrated that HAP2 was present at the conjugation junction, precisely where membrane fusion was taking place as was previously shown for *C. reinhardtii* and *P. berghei* (Cole et al., 2014).

Given that HAP2 is expressed in all seven mating types of *T. thermophila* and that pore formation appears to be initiated on both sides of the conjugation junction (see above, *Membrane Dynamics at Sites of Gamete Fusion*), one might predict that deletion of *HAP2* from one cell of a mating pair would have little-to-no effect on mating efficiency in the *Tetrahymena* system. Nevertheless, in crosses between genetically marked WT and *ΔHAP2* deletion strains, fertility was shown to decline by as much as 80% (Cole et al., 2014). This was consistent with subsequent findings that used flow cytometry and exchange of fluorescently-tagged cytosolic proteins between cells as a readout for pore formation in mating pairs (Pinello et al., 2017). In these latter studies, the number of pairs capable of exchanging dye was reduced by 80–90% in crosses between WT and *ΔHAP2* knockout strains (Pinello et al., 2017). Furthermore, the rate at which individual WT X *ΔHAP2* pairs formed pores was also significantly reduced, although the final level of protein exchange in the small percentage of cells that did form pores was essentially the same in WT X *ΔHAP2* and WT X WT crosses (Pinello et al., 2017). Taken together, these results suggested that successful mating between WT and *ΔHAP2* deletion strains is an all-or-none phenomenon with most pairs being unable to form pores (or, at least, a sufficient number of pores to allow measurable dye exchange). As argued below (see *Discussion*), the most plausible explanation for these findings is that cooperative interactions between the fusion machinery on both membranes of a mating pair is required for efficient pore formation to occur in *T. thermophila*.

The requirement for *HAP2/GCS1* in fertilization and its function downstream of gamete adhesion in a variety of different systems, clearly pointed to a role for HAP2 in membrane fusion. Nevertheless, until recently, large differences in primary amino acid sequence between *HAP2* orthologs of different species, together with an absence of homologies to known membrane fusogens left open questions regarding HAP2’s precise function. Those questions were answered using a variety of analytical approaches. First, HAP2 orthologs were shown to assume a 3-dimensional structure closely resembling class II membrane fusogens of enveloped viruses (Fédry et al., 2017; Pinello et al., 2017; Valansi et al., 2017). Second, biophysical studies demonstrated the ability of the HAP2 ectodomain, as well as predicted HAP2 fusion peptides to interact directly with model membranes (Fédry et al., 2017; Pinello et al., 2017). And finally, under appropriate conditions, ectopic expression of *A. thaliana* HAP2 ortholog was shown to be capable of mediating cell-cell fusion in cultured mammalian cells (Valansi et al., 2017). As described in the following section on protein structure, a large part of this work focused on HAP2 molecules from unicellular eukaryotes.

#### 3.4.1 Structural Requirements for HAP2 Function

**FIGURE 3:**
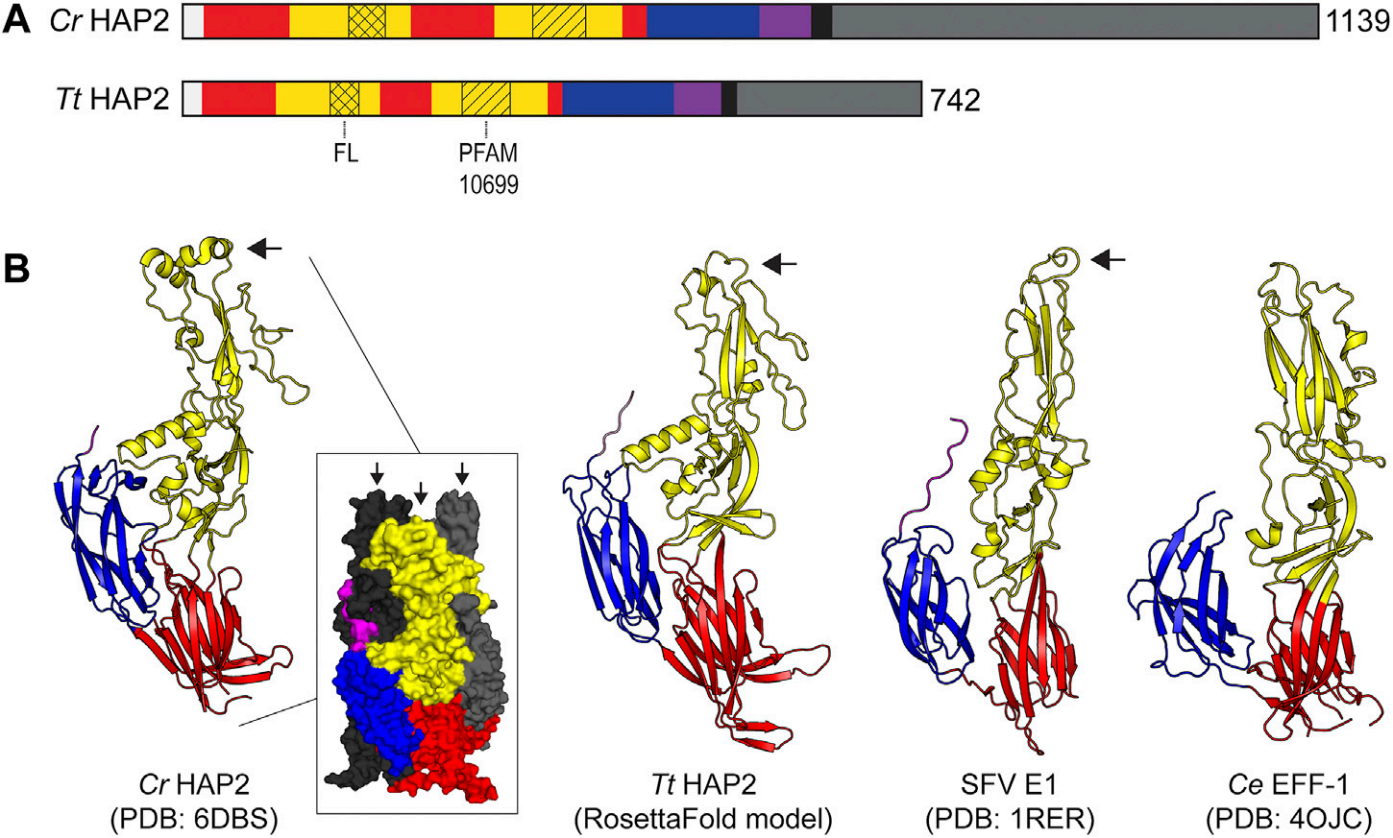
HAP2 Protein domains and structure. **(A)** Protein domain schematic showing the organization of HAP2 monomers from N′ to C′ terminus **(left to right)** from *T. thermophila* (Tt) and *C. reinhardtii* (Cr). The signal peptide is white; domain I is red; domain II is yellow; fusion loop (FL) is indicated with crosshatch; PFAM10699 (HAP2/GCS1 domain) is indicated with diagonal lines; domain III is blue; stem is purple; transmembrane domain is black; and, the cytoplasmic domain is gray. The number of amino acids in each protein is shown on the far right at the C′ terminus. **(B)** X-ray and model ectodomain protein structures showing the homology of HAP2 and other class II fusogens from viruses and cells. From left to right: the crystal structure of the *C. reinhardtii* HAP2 post-fusion trimer with one cartoon protomer **(left)** and a surface representation of the trimer (inset) with colored domains on the front protomer and the two back protomers in black and gray, respectively (Feng et al., 2018); a RosettaFold model protein structure of a *Tetrahymena* HAP2 monomer (Baek et al., 2021); a single protomer from the trimeric crystal structure of the Semliki Forest Virus E1 class II fusion protein (Gibbons et al., 2004); a single protomer from the trimeric crystal structure of the *C. elegans* class II cell-cell fusion protein EFF-1 (Perez-Vargas et al., 2014). Black arrows point to the fusion loops. Protein databank IDs (PDB) are shown below the images.

The basic structural features of HAP2/GCS1 orthologs from *Chlamydomonas* and *Tetrahymena* are shown in Figure 3A. In many ways, these proteins are representative of the HAP2/GCS1 family overall. Both are single-pass transmembrane proteins. Based on their primary sequence, they vary in size (the *C. reinhardtii* protein being somewhat larger at 1,139 amino acids, compared to 742 amino acids in the case of *T. thermophila* protein). They also share weak homology overall but are identifiable as cousins through homology at the so-called HAP2/GCS1 domain (PFAM 10699), a stretch of ∼50 amino acids in the extracellular domain that, with few exceptions (e.g. *Drosophila melanogaster*, is conserved across the HAP2/GCS1 family (Garcia, 2012; Fedry et al., 2018). Many orthologs, including the *Chlamydomonas* and *Tetrahymena* proteins, have a cysteine-rich, poly-basic stretch in the cytosolic region immediately following the transmembrane helix. Nevertheless, there is considerable variation in the size of HAP2/GCS1 cytosolic domains overall. For example, some species (e.g., *Chlamydomonas reinhardtii* and *Toxoplasma gondii*) have extended intracellular domains with 500 residues or more, while others have almost no cytoplasmic tail whatsoever (*Ichthyophthirius multifiliis* [a parasitic ciliate]; *Pediculus humanus corporis* [the human body louse]; and, *Trypanosoma cruzi* [the etiologic agent of Chagas disease] (Liu et al., 2015). Substitutions or deletions of the entire HAP2 cytoplasmic domain, or certain polybasic or potentially palmitoylated cysteine residues within the cytosolic domains of various HAP2/GCS1 orthologs have been shown to impact protein localization and/or function but those impacts appear to be different in different species and the functional role of the cytosolic domains is still under scrutiny (Mori et al., 2010; Wong et al., 2010; Liu et al., 2015; Pinello et al., 2017).

Early efforts to make sense of HAP2/GCS1 function relied heavily on mutational studies designed to link alterations in phenotype (either loss of fertility or failure to fuse) to either single amino acids or larger regions of protein structure (Mori et al., 2010; Wong et al., 2010; Liu et al., 2015; Pinello et al., 2017). While informative in some cases (for example, domain swaps between HAP2/GCS1 orthologs of related species showed some evidence of lineage-specificity in the extracellular domains of plant proteins (Wong et al., 2010)), this overall approach involved guess work, and as often as not, changes to the protein led either to a complete loss of expression (presumably due to protein misfolding) or failure of the protein to reach its correct destination within the cell for reasons that were not always easy to interpret.

Nevertheless, a breakthrough in our understanding of HAP2/GCS1 function came with the unraveling of the protein’s 3-dimensional structure using disparate approaches, namely, x-ray crystallography and structure homology modeling. In the latter case, algorithms that compare secondary structural elements in query sequences to known structures in the PDB Protein Databank (Raptor X; Phyre2; HHblits; LOMETS, etc.) were used by two laboratories to parse the structures of the HAP2/GCS1 ectodomains from *Tetrahymena* and *Arabidopsis* (Pinello et al., 2017; Valansi et al., 2017). At the same time, Felix Réy’s laboratory at the Pasteur Institute in collaboration with William Snell’s group at UT Southwestern Medical Center, solved the x-ray crystal structure of a recombinantly expressed version of the *C. reinhardtii* HAP2 ectodomain at 3.3Å resolution (Fédry et al., 2017). Both approaches led to the same conclusion—HAP2/GCS1 is a Class II fusion protein (CII). Improved structures of the *C. reinhardtii* protein, as well as a nearly complete and partial structure of the HAP2/GCS1 ectodomains from *Arabidopsis thaliana* and *Trypanosoma cruzi*, respectively (Fedry et al., 2018; Feng et al., 2018; Baquero et al., 2019), have validated this conclusion and shed additional light on the organization of these proteins particularly in the regions of the “fusion loops” (see below).

The striking architectural similarities between HAP2/GCS1 ectodomains from *C. reinhardtii* and *T. thermophila*, and comparable regions of the E1 glycoprotein of Semliki Forest Virus (a classic CII viral fusogen) and the Epithelial Fusion Failure protein one of *C. elegans* (a CII protein required for cell-cell fusion in nematode worms) are shown in Figure 3B (Gibbons et al., 2004; Perez-Vargas et al., 2014; Feng et al., 2018; Baek et al., 2021). As with other CII fusogens, the ectodomains of HAP2/GCS1 are comprised of three mostly beta-strand-containing globular regions (domains I, II and III) connected by a stem to a single transmembrane domain (Figures 3A,B). Importantly, all HAP2/GCS1 structures solved to date contain hydrophobic “fusions loops” between beta-strands c and d at the apical tip of domain II (Figure 3B, black arrow). Hydrophobic residues in these loop structures insert into the outer leaflets of apposed target membranes and are key functional determinants for membrane fusion present in all class II viral proteins (see below, *Models of HAP2/GCS1-mediated fusion*). Finally, consistent with the known behavior of class II fusogens (see below), purified HAP2/GCS1 ectodomains form trimers when interacting with liposomes or detergent and their solved structures reflect the trimeric, post-fusion conformation of each protein with its fusion loop and transmembrane domain positioned as they would be in the fused membrane (Fédry et al., 2017; Feng et al., 2018) (Figure 3B, left).

Along with these similarities, interesting differences between viral class II proteins and HAP2/GCS1 orthologs have also been noted. For example, the first crystal structure of the *Chlamydomonas* HAP2 ectodomain revealed that its long (39 amino acid) fusion loop is bisected by a salt bridge connecting an arginine residue at position 185 in the loop (R185) with a glutamic acid residue at position 126 (E126) (Fédry et al., 2017). These arginine and glutamic acid residues are highly conserved within the HAP2/GCS1 family but are notably absent in class II viral proteins and the *C. elegans* FF family of cell-cell fusogens. Moreover, the R185-E126 salt bridge in the algal protein appears to play a critical role in organizing the membrane interacting regions of HAP2/GCS1 to ensure fusion (Fédry et al., 2017; Fedry et al., 2018). Functional studies in *Chlamydomonas* showed that mutating the single R185 residue in the loop region completely blocked gamete fusion without disrupting either HAP2 protein expression or localization. Furthermore, *in vitro* studies with purified HAP2 ectodomains showed that interactions of trimers with liposomes (measured by their co-floatation on sucrose gradients) was strictly dependent on the presence of the R185 residue.

In subsequent work, a higher resolution (2.6 Å) structure of the *Chlamydomonas* HAP2 ectodomain provided additional information about the location, orientation, and structural relationships of key residues within the HAP2 fusion loop, including R185 (Feng et al., 2018). This new study revealed that residue R185 is highly dynamic within a stable carbonyl cage suggesting that it may serve as a flexible pivot point for the apical end of domain II relative to the trimer axis—a flexibility that could allow for adjustments in orientation and positioning of the fusion loops during the membrane fusion reaction. Furthermore, these results presented the possibility that this carbonyl cage could be one of the drivers of HAP2/GCS1 domain II conservation across species, since three of the four residues comprising the cage fall within this domain (Feng et al., 2018).

Despite the overall conservation of the arginine-glutamic acid salt bridge, fusion loops themselves appear to have undergone significant diversification within the HAP2/GCS1 lineage. Comparative studies of x-ray crystal structures of widely diverged HAP2/GCS1 orthologs from *Arabidopsis thaliana* and *Trypanosoma* cruzi have shown that whereas the plant protein has a single fusion loop with an amphipathic helix (dubbed αF) that juts towards the membrane surface, the parasite ortholog has three small nonpolar loops (Fedry et al., 2018). By contrast, the fusion loop region of the *Chlamydomonas* ortholog is unusually large and has two loops extending a total of three fusion helices containing hydrophobic residues (Feng et al., 2018). Whether these differences in structure reflect differences in the target membrane compositions with which these different species’ HAP2 proteins interact is unclear, however, the necessity of fusion loop helical domains has been demonstrated in membrane interaction studies *in vitro* as well as fertility assays *in vivo* (Feng et al., 2018; Baquero et al., 2019; Zhang et al., 2021).

#### 3.4.2 HAP2-dependent Fusion Requires Membrane Attachment and Trimerization

Studies of viral class II fusogens have laid the groundwork for our current understanding of how HAP2/GCS1 mediates gamete fusion. Following virus attachment and uptake into host cells, the low pH environment of the of the endosome triggers a dramatic intra- and intermolecular reconfiguration of the fusion proteins that promotes merger of the virus envelope with endosomal membranes (Wahlberg and Garoff, 1992; Lescar et al., 2001; Sánchez-San Martín et al., 2009). During this reconfiguration, the fusion loop at the tip of domain II becomes exposed and inserts into the endosomal membrane, creating a protein bridge between the two membranes (Hammar et al., 2003; Liu and Kielian, 2009). Individual fusion proteins then trimerize (Wahlberg et al., 1992; Liu and Kielian, 2009) and undergo a conformational change in which domain III (blue) folds back towards domain I and the lower part of domain II, creating a dimple in the apposed membranes. As fold back continues, the transmembrane anchor in the viral-membrane is brought into contact with the fusion loop in the endosomal membrane (Kielian and Rey, 2006; Sánchez-San Martín et al., 2009). The accompanying distortions of both membranes destabilize the lipid bilayers, leading to membrane fusion.

New work in *Chlamydomonas* has shown that 1) HAP2 forms trimers *in vivo* that are essential for fusion; 2) hydrophobic residues in the fusion loop are critical for enhancing trimerization; and, 3) mating structure adhesion is essential for HAP2 trimerization (Zhang et al., 2021). Similar to viral class II proteins (Wahlberg et al., 1992; Gibbons and Kielian, 2002; de Boer et al., 2012), it was found that some proportion of HAP2 trimers remains resistant to low heat and low concentrations of reducing agent (45°C, 10 mM DTT) making it possible to detect the presence of the trimer with SDS-PAGE and immunoblotting and follow the kinetics of trimer formation *in vivo* during the course of fertilization (Zhang et al., 2021). Within 10 min of mixing wild type *C. reinhardtii plus* and *minus* gametes, HAP2 trimers were readily detected on immunoblots. Interestingly, only a portion of total cellular HAP2 formed trimers (∼450 kDa), while the rest remained as two ∼150 kDa monomer bands. Site-directed mutagenesis of leucine residues (L310E and L448E) designed to interfere with hydrophobic interactions present at the trimer core inhibited the trimerization of recombinant HAP2 ectodomain *in vitro* and blocked gamete merger *in vivo*, indicating that trimer formation is essential for fusion. Importantly, HAP2 trimer formation was also found to specifically require gamete membrane adhesion. When *Chlamydomonas plus* gametes lacking the *Chlamydomonas* membrane adhesion protein FUS1 were mixed with *minus* gametes, cells underwent wild-type levels of ciliary agglutination, gamete activation, and even mechanical contacts at their mating structures, but failed to form HAP2 trimers and failed to fuse, showing that gamete membrane adhesion is required for HAP2 trimer formation *in vivo* (Zhang et al., 2021). Selective mutations of the large *Chlamydomonas* HAP2 fusion surface, including additive mutations of hydrophobic residues from one, two, or all three of the fusion helices, allowed the additional observation that after gamete membrane adhesion occurs, interactions of the *Chlamydomonas* HAP2 fusion loop with the target *plus* gamete membrane contribute to the formation of HAP2 trimers *in vivo*. These data were supported by *in vitro* studies showing that recombinant HAP2 ectodomains undergo spontaneous trimerization upon incubation with liposomes at neutral pH (Fédry et al., 2017; Baquero et al., 2019).

## 4 HAP2/GCS1 in Protozoan Parasites: Targets for Transmission-Blocking Vaccines and a Tool for the Identification of Cryptic Sexual Life Cycles

Parasitic protists are among the most important disease-causing agents of humans and animals, nevertheless vaccines targeting these agents have proven difficult to develop especially when compared their viral and bacterial counterparts (Bowman, 2014). The fact that immunity in response to natural infection often takes years to develop and may never afford complete or long-lasting protection are among the greatest challenges for the development of effective vaccines against parasitic agents. Parasites are masters of immune evasion and many of the difficulties associated with anti-parasite vaccine development can be ascribed to their complex lifecycles. For example, *Plasmodium falciparum*, an apicomplexan responsible for the most severe forms of human malaria, cycles between extracellular stages capable of expressing multiple, variant surface antigens over time as well as intracellular stages that escape antibody detection entirely. The development of effective subunit vaccines against such agents is akin to hitting a hidden, moving, and ever-changing target.

In addition to vaccines that are designed to block parasite infection and growth within the host, investigators are also focusing on transmission-blocking vaccines (TBV), which have the potential to interrupt the sexual phase of parasite’s life cycle that is required for disease transmission (Vogel, 2010). Like other eukaryotes, sex is an obligate part of the life cycle of many, if not most parasitic protists. Furthermore, antigens associated with certain parasites’ sexual development are often only expressed in an insect vector and can be highly conserved. Several such antigens, including HAP2, are considered promising candidates for transmission-blocking vaccine development. As discussed below, HAP2 orthologs are present in a wide range of parasitic protists where their essential function in gamete fusion is conserved. At the same time, the absence of *HAP2* genes in vertebrates lessens the potential for side-effects due to cross-reacting antibodies in immunized patients. Multiple studies investigating HAP2 as a possible vaccine antigen are now in progress and stand to benefit from structure/activity studies in model organisms designed to pinpoint regions of the protein most likely to bind neutralizing antibodies. Apart from vaccine development, studies of HAP2 expression have also begun to shed light on the cryptic sexual cycles of many parasitic protists. In either case, the preponderance of this work has been done with apicomplexans, distant relatives of ciliates and dinoflagellates, which are also among the most important parasites of humans and animals.

Coccidia comprise one of the largest subgroups of the Apicomplexa. In these organisms, sexual reproduction occurs in intestinal cells of infected animals with resultant oocysts being expelled in the feces and ingested by the next host. In the poultry industry, coccidia belonging to the genus *Eimeria* sicken birds and have a major impact on egg and meat production (Sharman et al., 2010). While vaccines for *Eimeria* are available, they often provide less than full protection against disease outbreaks. This and recently developed resistance to anti-coccidian chemotherapeutics underlines the need for improved control measures including new vaccines (Ahmad et al., 2016). One approach along these lines has been the development of CoxAbic^®^ (Wallach et al., 2008), a crude preparation of gametocyte antigens and one of the first examples of a transmission-blocking vaccine successfully employed against a parasite model (Wallach et al., 2008). Injection of this vaccine into breeding hens just before egg laying was found to reduce oocyst shedding and generate maternal antibodies that protected chicks from infectious challenge with three different *Eimeria* species through at least 8 weeks of age.

Following the success of this approach, specific sexual stage antigens produced as recombinant proteins are now being explored as *Eimeria* vaccine candidates (Jang et al., 2010). In 2015, RNA Seq analysis of *E. tenella* identified a variety of such antigens, including HAP2, which were found to be expressed in male microgametocytes in the caeca of infected chickens and not in asexual merozoite or sporozoite stages (Walker et al., 2015). This study revealed molecular aspects of fertilization in *Eimeria* that were not previously known and opens the door to testing HAP2 as a vaccine antigen for interrupting *Eimeria* transmission.

*Toxoplasma gondii*, another widely distributed coccidian and one of the most successful parasites on earth (Griffin et al., 2019) is also being explored as a target for transmission-blocking vaccine development. *T. gondii* has a broad host range infecting most warm-blooded animals and birds as intermediate hosts. Cats are the definitive hosts and become infected by ingesting prey containing tissue cysts, known as bradyzoites. Sexual reproduction occurs exclusively in the intestinal epithelia of infected animals and gives rise to oocysts that are shed in the feces. As a disease agent, *Toxoplasma* has a significant economic impact in the sheep industry where it causes abortion (Innes et al., 2009) and can be highly pathogenic in wildlife species such as sea otters (Miller et al., 2004). In humans, *T. gondii* generally causes mild-to-asymptomatic disease in healthy individuals but can have devasting effects in immunocompromised adults, as well as children infected *in utero* during pregnancy. The ability of *T. gondii* to persist for long periods both in the environment and within the host makes this agent especially difficult to control, and while veterinary vaccines have been developed that reduce the formation of tissue cysts (Innes et al., 2009; Zhang et al., 2013), a strategy to lower the prevalence of *Toxoplasma* oocysts in the environment would be highly desirable. Toward that end, recent progress has been made in understanding the expression and regulation of sexual stage antigens in *T. gondii*, including HAP2.

In 2020, Farhat et al. discovered a MORC-driven transcriptional switch that controls *T. gondii* development and sexual commitment (Farhat et al., 2020). Through the assembly of histone deacetylase, HDAC3, and HAP2-related transcription factors, MORC was found to repress the transcription of HAP2 and a broad set of other sexual stage genes in non-sexual stages of *T. gondii*. Depletion of MORC allowed the release of this transcriptional repression and the expression of *HAP2* (Farhat et al., 2020). While the HAP2 protein has yet to be detected or localized to specific stages of the *T. gondii* life cycle, consistent with previous studies in coccidian *E. tenella* (Walker et al., 2015), *HAP2* RNA transcripts were found to increase in abundance during parasite development in the intestinal tissue of infected cats (Ramakrishnan et al., 2019).

With the idea that HAP2 plays a critical role in fertilization, *T. gondii*, strains lacking the *HAP2* gene were generated and tested as potential live attenuated vaccines (Ramakrishnan et al., 2019). Following administration to cats, *HAP2* deletion strains produced only small numbers of misshapen oocysts that showed no evidence of meiosis or diploidy and failed to sporulate. This would strongly suggest that HAP2 is required for fertilization in *T. gondii* and puts to rest earlier speculation regarding the role of fertilization in the development of infectious oocysts (Ferguson, 2002). When tested as live vaccines, the *ΔHAP2* deletion strains, as might be expected, did not prevent systemic infection of immunized cats challenged with virulent wild-type parasites (Ramakrishnan et al., 2019), however, they did completely block the production of infectious oocysts in these animals providing an exciting proof-of-principle that a transmission-blocking vaccine for *T. gondii* is within reach. As an alternative to rationally attenuated vaccines (which require growth of large numbers of parasites), one could easily envisage the use of recombinant subunit or nucleic acid vaccines encoding the HAP2 protein.

In addition to studies with *Eimeria* spp. and *Toxoplasma*, HAP2 expression has been documented in several other coccidian species including *Cystoisospora suis* and *Cryptosporidium parvum* (Feix et al., 2020; Lippuner et al., 2018)*.* In *Cystoisospora suis*, an agriculturally important pathogen of swine, cell-free culture conditions were identified that allow progression of asexual merozoites into sexual stages of the parasite through the oocyst stage *in vitro*. *HAP2* transcripts were found to be upregulated during this progression (Feix et al., 2021) leading to interest in HAP2 as a possible transmission-blocking vaccine for this species as well. By contrast, *HAP2* transcripts were found to be present at similar levels in all stages of the life cycle of *Cryptosporidium parvum* (Lippuner et al., 2018). While somewhat unexpected given the pattern of HAP2 transcription in other organisms, certain species, such as the alga *Gonium pectorale*, have been found to the control the stage-specific expression of HAP2 post-translationally (Kawai-Toyooka et al., 2014), and the levels of HAP2 protein within different stages of *C. parvum* are still not known.

In addition to the coccidians, HAP2 is being actively studied in Haemosporidia (similar to Aconoidasida), an important group of apicomplexan parasites that cycle between vertebrate and arthropod hosts. Within this group, *Babesia spp*. and *Plasmodium spp*. are hugely consequential. The genus *Babesia* is comprised of >100 species that cause tick-borne illness in humans and animals and result in significant economic losses particularly in the cattle industry (Griffin et al., 2019). Recent studies in *Babesia bovis* have shown that *HAP2* gene expression occurs within tick midgut and not in blood-stage parasites (Hussein et al., 2017). Consistent with this, deletion of the *HAP2* gene blocked morphological development of gametic stages and prevented expression of the 6-cys family member *A* and *B* genes, which are normal markers of sexual stage parasites in the tick midgut (Alzan et al., 2016; Hussein et al., 2017). In 2017, studies of *HAP2* expression in *B. bigemina* (another species responsible for bovine babesiosis) resulted in similar findings and, more importantly, showed that antibodies against conserved HAP2 peptides significantly reduced the *in vitro* formation of zygotes from sexual forms (Camacho-Nuez et al., 2017). Although multiple wildlife species can serve as reservoirs for *Babesia spp*., the development of transmission-blocking vaccines targeting HAP2 could be useful for reducing parasite prevalence in endemic areas where domesticated cattle herds routinely graze.

The genus *Plasmodium* can also infect humans and animals but is most well known in the context of human malaria, a disease responsible for ∼400,000 deaths (the majority in children) and over a million cases each year primarily in sub-Saharan Africa (Griffin et al., 2019). Of the five *Plasmodium* species that infect humans, *P. falciparum* is the most important in terms of overall morbidity and mortality. Despite decades of effort, only a single vaccine targeting *P. falciparum*, viz. Mosquirix™ (GlaxoSmithKline), has been recommended by the World Health Organization for widespread use. This 3-dose recombinant subunit formulation contains the major surface antigen on infectious sporozoites, namely, CSP, and provides ∼34% efficacy in preventing severe disease in children aged 5–17 months (RTS,S Clinical Trials Partnership, 2014; Laurens, 2020). A more recent, R21/Matrix-M vaccine, which also targets CSP and uses a different adjuvant, appears more promising (Datoo et al., 2021). The latter vaccine is undergoing phase three clinical trials (University of Oxford, 2021) but is not yet approved.

The expression of variant surface antigens at different stages of the parasite life cycle is among the most important reasons for the failure of vaccines targeting *P. falciparum*. Nevertheless, parasite transmission requires an obligate sexual stage in which male and female gametocytes are produced in the human and then transferred to female Anopheles mosquitoes when they take a blood meal. Gametocytes complete development in the mosquito midgut and then undergo fertilization. Because these final steps in sexual development occur only in the insect vector, *Plasmodium* proteins expressed after ingestion by mosquitos are not subject to selective pressure from the vertebrate immune system (and therefore less subject to variation) but are nevertheless exposed to antibodies taken up with the blood meal. Indeed, there is now considerable evidence for the effectiveness of immunization with sexual stage antigens in blocking parasite transmission within closed laboratory settings demonstrating proof-of-concept for the use of this approach in the field (Blagborough et al., 2013).

The presence of *Plasmodium* HAP2 orthologs was recognized in 2006 (Mori et al., 2006), and it was not long after that *HAP2* was shown to be essential for fertilization of *P. berghei* (Liu et al., 2008). In the latter case, *HAP2* gene disruption had no effect on either exflagellation or the adherence male and female gametes, but completely blocked gamete fusion and ookinete development (Liu et al., 2008). By contrast, macrogametes lacking *HAP2* were fully capable of fertilization and ookinete development following interaction with wild type microgametes (Hirai et al., 2008; Liu et al., 2008). These findings spurred considerable interest in HAP2 as a candidate antigen for the development of transmission-blocking vaccines for *Plasmodium* (Sinden et al., 2012). In promising studies, Angrisano and co-workers have shown that immunization of mice with a short, 18-residue polypeptide encoding the *Plasmodium berghei* HAP2 fusion loop, elicited specific humoral antibody responses capable of blocking fertilization *in vitro* by up to 89.9% and transmission *in vivo* by up to 58.9% when mosquitoes were fed on immunized mice (Angrisano et al., 2017). Furthermore, a significant, dose-dependent reduction in the number of oocysts present in the mosquito midgut was seen when antibodies against the *P. falciparum* HAP2 fusion loop were mixed with infected blood from African donors and then fed to mosquitoes in standard membrane feeding assays (Angrisano et al., 2017). By combining immunogenic peptides with recombinant protein-based vaccine formulations now being tested for malaria (Datoo et al., 2021; Jelínková et al., 2021) it may be possible to reduce parasite prevalence in endemic areas through reduced transmission while at the same time protecting individuals against disease.

Lastly, HAP2 orthologs have been identified in the Kinetoplastid parasites of humans in the genera *Trypanosoma* and *Leishmania*. While it is unclear whether fertilization/sexual reproduction is obligatory for infectious transmission of these parasites, *Trypanosomes* undergo a sexual stage in tsetse fly or triatomine insect vectors as evidenced by genetic exchange (Jenni et al., 1986; Gaunt et al., 2003), cytoplasmic mixing between parasites (that is cell-cell fusion) (Gibson et al., 2008), and the expression of conserved meiosis-specific genes prior to cell-cell fusion (Peacock et al., 2011, 2014). Recent single-cell RNA-seq studies have demonstrated that *T. brucei* HAP2 is one of a cluster of gamete-specific genes upregulated in the salivary glands of infected tsetse flies (Hutchinson et al., 2021), and YFP-tagged HAP2 has been shown to be expressed in parasites isolated from tsetse fly salivary glands (Castellanos, 2018). As noted above, the crystal structure of domain II of *T. cruzi* HAP2 has also been solved and displays overall conservation of structure when compared to other the class II fusion proteins, but also has substantial differences from *Chlamydomonas* and *Arabidopsis* HAP2 in the arrangement and structure of its membrane interaction motif (Fedry et al., 2018).

A HAP2/GCS1 ortholog has also been shown to be present in *Leishmania spp*. (Hirai et al., 2008; Liu et al., 2008), and studies of its expression and function have begun to offer unique insights as well as a new tool to study the parasites’ cryptic sexual cycle. *Leishmania* promastigotes experience a meiosis-like genetic exchange during their development in the Phlebotomine sandfly vector (Akopyants et al., 2009; Rougeron et al., 2010; Inbar et al., 2019), but less is known about the cellular interactions that accompany this process. Recently, however, DNA damage-induced cell stress has been shown to elicit upregulation in the expression of HAP2 and other conserved sexual transcripts, as well as an increase the efficiency of inter- and intra-specific genetic hybridization of *Leishmania* spp. *in vitro* (Louradour et al., 2021). Indeed, it is now possible to use the expression of an mNeonGreen-tagged HAP2 transgene as a marker for *L. tropica* promastigote mating competence. Separation of cells using fluorescence activated cell sorting has made it possible to examine matings between parasites that either do or do not express HAP2, and only promastigotes expressing HAP2 were found to be capable of hybridization. Reminiscent of work in *Tetrahymena*, these studies also showed that while the presence of HAP2 was required in only one of the two parental populations for genetic hybrids to form, crosses in which both parental lines expressed HAP2 showed much higher frequencies of hybrid formation (Louradour et al., 2021). Overall, this approach offers fundamentally new opportunities for dissecting the facultative sexual stage of *Leishmania* parasites.

## 5 Discussion

Despite everything we have learned about the role of HAP2/GCS1 in gamete fusion, there are still many unanswered questions regarding the HAP2-dependent fusion machinery, its evolutionary history, and its potential application in blocking fertility in parasites or other species. Regarding the fusion machinery itself, like viral CII fusogens, HAP2 must localize to specific sites on the plasma membrane and then assemble into a trimer to ensure that fusion occurs. Elucidation of the HAP2 pre-fusion conformation and a further understanding of the mechanisms that trigger its transition to a trimeric state will require additional work. As noted here, there is abundant evidence that HAP2/GCS1 does not act alone. Beyond membrane attachment, there are likely additional factors that are important in localizing HAP2 to sites of gamete fusion as well as promoting fusion itself. These could include other proteins such as actin, or DMP8/9-like proteins which adopt a faciliatory role in gamete fusion in *Arabidopsis* (Takahashi et al., 2018; Cyprys et al., 2019), as well as specific lipids that may accommodate HAP2/GCS1 fusion loops or promote membrane curvature itself.

**FIGURE 4:**
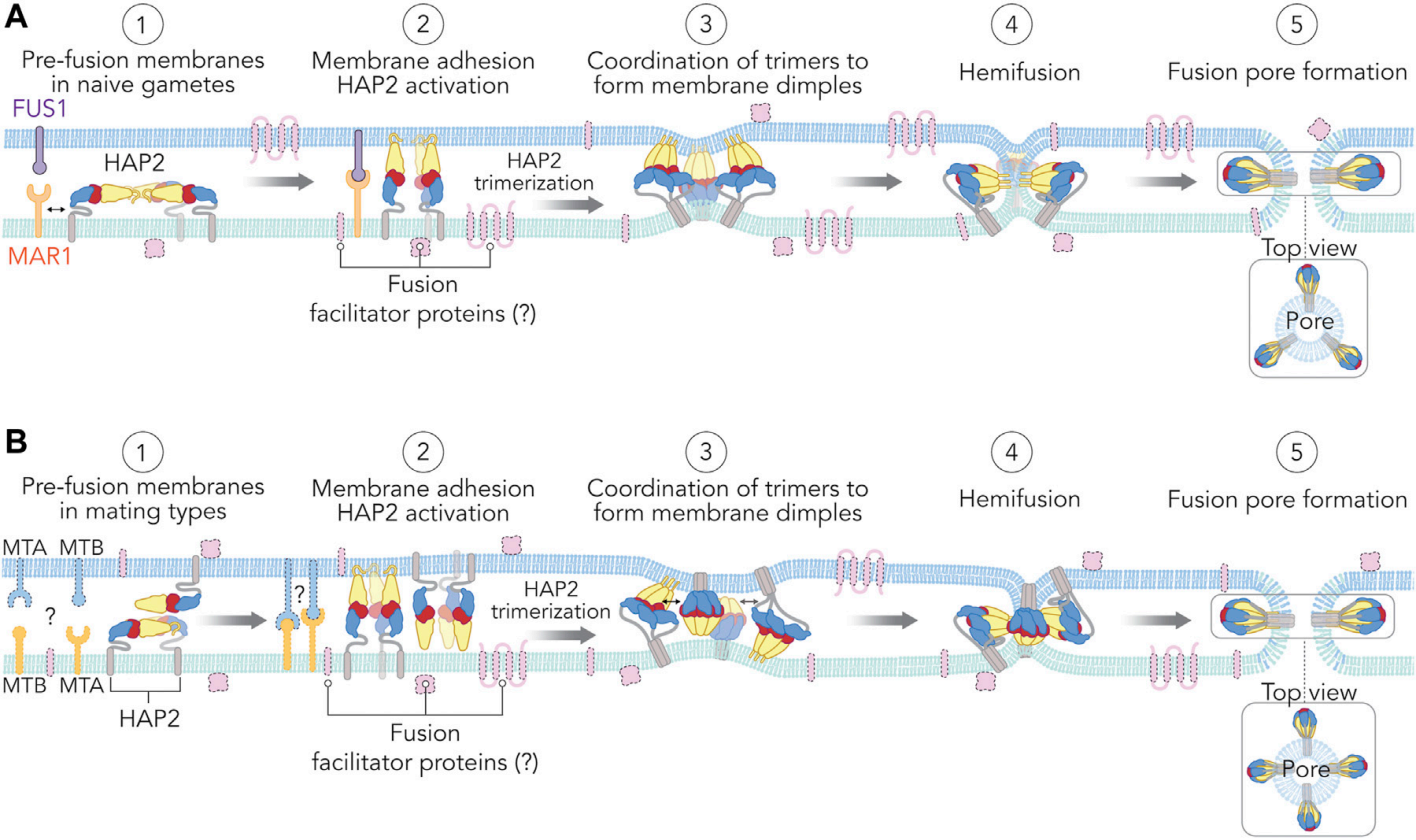
Model molecular mechanisms for the **(A)** *Chlamydomonas* and **(B)** *Tetrahymena* gamete membrane fusion reactions. **(1)** Before fusion, HAP2 is expressed on the *Chlamydomonas minus* gamete mating structure, and membranes at the developing conjugation junction of *Tetrahymena* in an as yet unknown pre-fusion state. Both species strongly upregulate HAP2 expression after mixing of different mating types. The cytoplasmic domains of some proteins are not shown. Also, other conserved or species-specific fusion facilitator proteins (pink) that have not yet been identified may actively participate in this process. **(2)** In *Chlamydomonas,* FUS1-MAR1 mediated membrane adhesion activates HAP2 out of its pre-fusion state on naive *minus* gametes, driving HAP2 fusion loop interactions with the *plus* gamete membrane and trimer formation. In *Tetrahymena* the membrane adhesion proteins are unknown, but it is possible that MTA and MTB proteins are involved in this recognition event, and similarly activate HAP2. **(3)** During conformational changes, the fusion loops and transmembrane domains of HAP2 proteins anchored in both membranes helps pull the two lipid bilayers close together. A circular coordination of several HAP2 trimers undergoing such changes is expected to form dimples in the apposed membranes. *Chlamydomonas* HAP2 trimers bridge the two membranes with their fusion loops anchored in the target *plus* gamete membrane, whereas *Tetrahymena*, having HAP2 protein expressed and functional on the membranes of both mating types, could undergo a different coordination of HAP2 trimers that contributes to its fusion efficiency. **(4)** As HAP2 conformational changes continue to fold domain III (blue) back against the domain II (yellow) the two membranes are pulled even closer together, helping to initiate hemifusion, a lipid mixing event between the outer leaflets of the two bilayers. **(5)** Completion of HAP2 trimerization induces full mixing of both leaflets of the lipid bilayer and creates membrane fusion pore with stable post-fusion HAP2 homotrimers fastened in the fused membrane by their fusion loops and transmembrane domains. In this model, for simplicity the fusion pore is encircled by three or four trimers in a top-down view, but in reality, it is not known how many HAP2 trimers are necessary for fusion pore formation in any species.

Regardless of any hypothetical requirement for additional facilitators, a basic model for HAP2/GCS1-mediated gamete fusion emerges from work on *Chlamydomonas* (Figure 4). As indicated earlier, HAP2 in its pre-fusion conformation is expressed in *minus* gametes at the site of the nascent mating structure and is further upregulated during interactions with *plus* gametes. FUS1-MAR1 mediated membrane adhesion then facilitates HAP2 activation, allowing interaction of its fusion loops with membranes on *plus* gametes and driving trimerization of the protein (Figures 4A-1,2). Presumably, coordinated foldback of several HAP2 trimers then leads to the formation of dimples on apposed membranes where the multiple fusion loops and transmembrane domains congregate (Figures 4A-3). As foldback continues, the two apposed membranes are pulled even closer, helping overcome the hydration barrier between the two bilayers and initiating hemifusion, the mixing of the outer leaflets of the two bilayers (Figures 4A-4). As transmembrane domains and fusion loops come together, full mixing of the two leaflets occurs and creates a membrane fusion pore (Figures 4A-5). In *Chlamydomonas*, the pore(s) expands quickly to completely fuse the two gamete cells.

While this model is straightforward and comports with a vast body of work on viral CII proteins where fusion is driven unilaterally from one membrane, data on HAP2/GCS1-mediated gamete fusion in *Tetrahymena*, along with studies of AFF-1/EFF-1-mediated cell-cell fusion in *C. elegans* force a consideration of alternative models in instances where efficient pore formation requires that fusogens be present on apposed membranes. As described earlier, while a small percentage of *Tetrahymena* mating pairs can undergo fusion when HAP2 is expressed unilaterally on one membrane, the low efficiency of pore formation in crosses of wildtype cells with *ΔHAP2* deletion partners strongly suggests that some type of bilateral interaction occurs between mating cells that is HAP2-dependent. Although one can only speculate as to what those interactions might be, it is reasonable to infer they involve either 1) heterotypic interactions between HAP2 and some hypothetical receptor(s) on apposed membranes that allows pores to form more readily (for example by enhancing membrane adhesion); 2) homotypic interactions between HAP2 monomers/dimer/trimers across apposed membranes that promote the initiation and/or opening of fusion pores between cells; or, 3) some combination of hetero- and homotypic interactions. Before speculating further, it is worth noting that massive overexpression of HAP2 in wild type cells (WT-OE) using a high-copy ribosomal DNA vector paired with a robust cadmium-inducible promoter, failed to increase the percent fusion observed in crosses between WT-OE and *ΔHAP2* deletion strains (Pinello et al., 2017). The inability of HAP2 overexpression to rescue or compensate for the lack of HAP2 on the apposed membrane would argue that the low fusion efficiency seen in these crosses is likely not due to in insufficient density of HAP2 on one membrane. Indeed, these data reinforce the idea that fusion pore formation is an all-or-none phenomenon since the opening of even a small number of pores in such crosses might be expected to rescue fusion efficiency of the *ΔHAP2* partner through the transfer of *HAP2* mRNA from the wildtype cell to its *ΔHAP2* partner.

Along with these observations, studies with the class II fusogens AFF-1 and EFF-1 from *C. elegans* also support the idea that CII fusogens can interact across membranes. In mosaic animals containing mixtures of cells that retained or lacked the *eff-1* gene, cell-cell fusion only occurred between cells that contained the gene (Podbilewicz et al., 2006). Furthermore, ectopically expressed AFF-1 and EFF-1 were capable of driving fusion of heterologous cells and, in some cases, could substitute for viral fusogens in pseudotyped virus infection assays, but only when the proteins were expressed in adjacent cells (in the case of cell-cell fusion) or target cells (in the case of virus fusion assays) (Podbilewicz et al., 2006; Sapir et al., 2007; Avinoam et al., 2011; Perez-Vargas et al., 2014). Given that AFF-1 and EFF-1 lack bona fide fusion loops and have no obvious way to generate motive force on apposed membranes, a requirement for bilateral (*trans*-) interactions between these proteins makes sense and various models for AFF-1/EFF-1-mediated pore formation involving monomer-dimer as well as trimer-trimer interactions have been proposed (Podbilewicz, 2014; Zeev-Ben-Mordehai et al., 2014). Similarly, studies with *Arabidopsis* HAP2 (Valansi et al., 2017) indicate that bilateral interactions are required for syncytia formation when the plant protein is expressed ectopically in mammalian cells despite the fact that it functions unilaterally during fertilization and appears to have a functional fusion loop (Johnson et al., 2004; Mori et al., 2006; Fedry et al., 2018).

There is considerable evidence that *Tetrahymena* HAP2/GCS1 also has a functional fusion loop, although substitution of an alanine residue for the highly conserved arginine expected to play a critical role in stabilizing the loop (see above, *Structural requirements for HAP2/GCS1-mediated fusion*) had no effect on fusion in crosses between wildtype and mutant (HAP2-R164A) *Tetrahymena* strains (Pinello et al., 2017). While this could be interpreted to mean that *T. thermophila* HAP2 does not have (or does not require) a fusion loop, it is entirely possible that the wildtype protein on one mating partner can rescue an otherwise defective HAP2-R164A on its mating partner through *trans*-interactions between HAP2 proteins on apposed membranes. To test that idea, it will be necessary to examine the effects of the HAP2-R164A mutation expressed in both cells of a mating *Tetrahymena* pair. Crosses between HAP2-R164A mutant cell lines would be expected to generate wildtype levels of fusion (if *T. thermophila* HAP2 can function in the absence of a fusion loop), while the same crosses would be expected to completely block fusion (if *T. thermophila* HAP2 requires a functional fusion loop in at least one mating partner). Certainly, the latter outcome would argue the importance of bilateral *trans*-interactions between HAP2 proteins on both cells of a mating pair and necessitate a model for HAP2-mediated fusion in *Tetrahymena* that accommodates both *trans*-interactions of the fusogen as well as HAP2 fusion loop interactions with the membrane. More generally, these types of studies raise the possibility that class II fusion proteins can blend different aspects of these underlying unilateral and bilateral activities depending on the context in which they are expressed. A model consistent with the idea that *Tetrahymena* HAP2 contains a functional fusion loop and can function bilaterally across membranes is shown in Figure 4B. Certainly, there is no *a priori* reason that the expression of HAP2, or any other gamete fusogen, should be restricted to a given mating type. Indeed, from an evolutionary perspective, the expression of HAP2/GCS1 in both mating partners in the case of *Tetrahymena* and other species may be a reflection of an ancestral isogamous state that was discarded in sexually dichotomous organisms (Mori et al., 2006; Cole et al., 2014; Kawai-Toyooka et al., 2014; Okamoto et al., 2016).

On a broader level, while the relationship between eukaryotic and viral class II fusogens is certainly intriguing and has obvious implications for the origins of eukaryotic sex, perhaps more pertinent to this review is whether HAP2/GCS1 has any role in fertilization in vertebrates. To date, database searches for HAP2/GCS1-like sequences in vertebrate genomes have come up short. Given the weak homologies between bona fide HAP2/GCS1 orthologs this may not be entirely surprising, however, it is just as likely that the corresponding gene was lost in the lineages leading to vertebrates. Given their conserved 3-dimensional organization, comparisons between known HAP2/GCS1 structures and predicted structures of protein-coding sequences within vertebrate genomes might yield useful information along these lines. Additional structural features conserved among HAP2/GCS1 orthologs might also serve as useful markers in this regard (Fedry et al., 2018).

Finally, on a more practical level, studies of parasitic protists have now made clear the utility of HAP2/GCS1 both as a vaccine target, and as marker for cryptic sexual activity in various species. Continued development of HAP2/GCS1 as an immunogen, along with further proof-of-concept that transmission-blocking vaccines (either in human or veterinary medicine) can be effective in natural settings are much anticipated. *Eimeria* and *Babesia* are perhaps the best models in which to test the effectiveness of such vaccines on reducing parasite prevalence in open agricultural environments. Along with the discovery that antibodies to the *Plasmodium* HAP2 fusion loop alone can generate a transmission blocking effect in mice and humans (Angrisano et al., 2017), it will also be important to identify a range of other possible HAP2 epitopes that can elicit neutralizing antibodies in target species. Useful in this regard would be further research to identify the pre-fusion structure of HAP2/GCS1 as well as interacting partners that could themselves be targets for vaccination, along with strategies to improve vaccine potency and delivery. For example, the use mRNA-based vaccines or repeat arrays of short immunogenic peptides as were used in a recent malaria vaccine targeting the circumsporozoite protein (Jelínková et al., 2021) could potentially bypass the need to make full-length or partial versions of the HAP2/GCS1 protein which can be difficult using conventional recombinant protein expression platforms. With further success along these lines, it may soon be possible to develop new types of bivalent parasitic vaccines that include both an immunogen designed to protect the vaccinated individual from severe disease, along with a HAP2/GCS1-type immunogen to block parasite transmission and provide a broader level of protection across a community. It is possible that such a vaccine approach might allow a greater reduction in overall disease prevalence with fewer individuals needing to be vaccinated.
